# Edging on Mutational Bias, Induced Natural Selection From Host and Natural Reservoirs Predominates Codon Usage Evolution in Hantaan Virus

**DOI:** 10.3389/fmicb.2021.699788

**Published:** 2021-07-02

**Authors:** Galal Ata, Hao Wang, Haoxiang Bai, Xiaoting Yao, Shiheng Tao

**Affiliations:** ^1^State Key Laboratory of Crop Stress Biology in Arid Areas, College of Life Sciences, Northwest A&F University, Xianyang, China; ^2^College of Veterinary Medicine, Northwest A&F University, Xianyang, China

**Keywords:** mutational bias, natural selection, codon usage, evolution, hantaan virus

## Abstract

The molecular evolutionary dynamics that shape hantaviruses’ evolution are poorly understood even now, besides the contribution of virus-host interaction to their evolution remains an open question. Our study aimed to investigate these two aspects in Hantaan virus (HTNV)—the prototype of hantaviruses and an emerging zoonotic pathogen that infects humans, causing hemorrhagic fever with renal syndrome (HFRS): endemic in Far East Russia, China, and South Korea—via a comprehensive, phylogenetic-dependent codon usage analysis. We found that host- and natural reservoir-induced natural selection is the primary determinant of its biased codon choices, exceeding the mutational bias effect. The phylogenetic analysis of HTNV strains resulted in three distinct clades: South Korean, Russian, and Chinese. An effective number of codon (ENC) analysis showed a slightly biased codon usage in HTNV genomes. Nucleotide composition and RSCU analyses revealed a significant bias toward A/U nucleotides and A/U-ended codons, indicating the potential influence of mutational bias on the codon usage patterns of HTNV. Via ENC-plot, Parity Rule 2 (PR2), and neutrality plot analyses, we would conclude the presence of both mutation pressure and natural selection effect in shaping the codon usage patterns of HTNV; however, natural selection is the dominant factor influencing its codon usage bias. Codon adaptation index (CAI), Relative codon deoptimization index (RCDI), and Similarity Index (SiD) analyses uncovered the intense selection pressure from the host (Human) and natural reservoirs (Striped field mouse and Chinese white-bellied rat) in shaping HTNV biased codon choices. Our study clearly revealed the evolutionary processes in HTNV and the role of virus-host interaction in its evolution. Moreover, it opens the door for a more comprehensive codon usage analysis for all hantaviruses species to determine their molecular evolutionary dynamics and adaptability to several hosts and environments. We believe that our research will help in a better and deep understanding of HTNV evolution that will serve its future basic research and aid live attenuated vaccines design.

## Introduction

Hantaviruses are rodent-borne pathogens that compose the genus *Orthohantavirus*, family *Hantaviridae*, in the order *Bunyavirales*^1^. Hantaan virus (HTNV), one of the Old World Hantaviruses and the prototype of all hantaviruses, infects humans, causing hemorrhagic fever with renal syndrome (HFRS): a serious disease with severe symptoms including fever, renal dysfunction, hemorrhagic manifestations, and shock with mortality rate vary from 0.3 to 12% (Jonsson et al., 2010; Krautkrämer et al., 2013; Avšič-Županc et al., 2019). HTNV, as one member of the genus *Orthohantavirus*, is a negative sense, single-stranded RNA virus with a tripartite genome that comprises large (L), medium (M), and small (S) segments: named by their size. S segment encodes the nucleocapsid (N) protein, while M and L segments encode the precursor (GPC) for two viral surface glycoproteins (G1 and G2) and viral polymerase, respectively, (Khaiboullina et al., 2005; Muyangwa et al., 2015; Liu et al., 2020). HTN, endemic in Eurasia (Far East Russia, China, and South Korea), is mainly related to the spread of its natural reservoirs: the striped field mouse (*Apodemus agrarius*) and the Chinese white-bellied rat (*Niviventer confucianus*) (Wang et al., 2000; Avšič-Županc et al., 2019).

The majority of organisms use the standard genetic code in protein translation. The genetic code comprises 64 codons, 61 codons translated into 20 amino acids, and the remaining three are stop codons. As the genetic code is degenerate, several codons can code the same amino acid, except methionine, and tryptophan, termed synonymous codon usage. The synonymous codons are unevenly used, and for the same amino acid, some codons might be prioritized over others. This uneven use of codons is termed codon usage bias (Martín et al., 1989; Plotkin and Kudla, 2011; Chaney and Clark, 2015). Biased codon usage is a crucial measure of the genome evolution and has been reported in most organisms, from prokaryotes to eukaryotes and viruses (Sharp et al., 1988; Plotkin and Kudla, 2011; Belalov and Lukashev, 2013; Chaney and Clark, 2015). Several factors were found to influence the biased codon choices: genetic drift, mutation pressure, natural selection, amino acid composition, secondary protein motifs, protein’ hydrophobicity and hydrophilicity, transcriptional factors, and external environment (Bulmer, 1991; Butt et al., 2016; Velazquez-Salinas et al., 2016b; Rahman et al., 2018; Labella et al., 2019; Yao et al., 2020). Nevertheless, the main factors that account for codon usage variation among different organisms are natural selection and mutation pressure coupled with genetic drift (Bulmer, 1991; Musto, 2016; Labella et al., 2019).

Regarding viral genomes, codon usage is a driving force in their evolution (Dutta et al., 2020). Some researchers have proposed that mutational bias is the primary determinant of the codon usage patterns in human RNA viruses (Jenkins and Holmes, 2003; Nasrullah et al., 2015; van Hemert et al., 2016; Tort et al., 2020), while others have reported the dominant influence of natural selection (Wang et al., 2016; Baha et al., 2019; Khandia et al., 2019; Luo et al., 2020). As parasitic organisms, viruses have some characteristics in their genomes different from prokaryotes and eukaryotes, for instance, relying on their hosts’ translational machinery for gene expression. This virus-host interaction influence the overall viral survival, adaptation, evasion of the host immune response, and evolution (Nasrullah et al., 2015; Rahman et al., 2017; Nguyen et al., 2021).

Phylogeneticists argued for and against the role of host-pathogen co-divergence in the evolution of hantaviruses that kept this notion an open question (Hughes and Friedman, 2000; Plyusnin and Morzunov, 2001; Ramsden et al., 2008, Ramsden et al., 2009; Kang et al., 2009; Li et al., 2020). A previous *in silico* study reported the codon usage analysis in different hantaviruses segments, where the authors proposed the dominant effect of mutation pressure on shaping hantaviruses codon usage bias (Sankar et al., 2015). However, due to notable limitations in this study—for instance, they depended mainly on the dinucleotide composition analysis to derive their hypothesis, ignoring the investigation of the virus-host interaction—we saw that specific, deep, and comprehensive research is still needed to test their hypothesis. Furthermore, except for Sanker et al., no other studies reported the codon usage bias in hantaviruses, leaving a gap in understanding their molecular evolutionary processes and adaptability to several hosts and environments.

For a more profound investigation of these two aspects, among several pathogenic species of hantaviruses, we specified HTNV—the prototype of hantaviruses and has the largest available number of strains with complete genomic sequence^2^ —with a comprehensive, stepwise phylogenetic-dependent codon usage analysis. For the first time, our report revealed the molecular evolutionary dynamics in one of the most important species of genus *Orthohantavirus* that will help in a better and deep understanding of its gene expression regulation and further aid live attenuated vaccines design (Velazquez-Salinas et al., 2016a). Also, it opens the door for more hantaviruses species-specific codon usage analysis to investigate the evolutionary pressures controlling its evolution and adaptability to several hosts and environments.

## Materials and Methods

### Data Description

All available HTNV strains with complete genomic sequences (L, M, and S segments) were collected and downloaded from the Virus Pathogen Resource database (ViPR, RRID:SCR_012983, accessed on September 8, 2020)^2^ and the National Center for Biotechnology (NCBI, GenBank, RRID:SCR_002760)^3^, respectively. The Open Reading Frames (ORFs) for each strain were obtained by Lasergene SeqBuilder Pro (DNASTAR): Lasergene Core Suite, RRID:SCR_000291, version 17.2.1). Sequence similarity check based on the concatenated genome (L + M + S) was performed for all strains (one against all) by Supermatcher online server^4^. Only one strain was retained from every two or more strains with 100% sequence similarity, resulted in 95 strains with genetic diversity ranging from 77 to 99.9%. Supplementary Table 1 shows all strains information. In addition, host, human (*Homo sapiens*, HS), and natural reservoirs, striped field mouse (*A. agrarius*, APO) and Chinese white-bellied rat (*N. confucianus*, NC), codon usage data were retrieved from the Codon and Codon-Pair Usage Tables database (CoCoPUTs, RRID:SCR_018504)^5^ (Alexaki et al., 2019).

### Recombination and Phylogenetic Analyses

Potential HTNV recombinant sequences were detected by Recombination Detection Program software (RDP, RRID:SCR_018537, version Beta 5.05) that implements an extensive, powerful array of methods to detect and visualize recombination events in virus genome sequence alignments (Martin et al., 2015). The default settings were applied for all analyses. Phylogenetic analysis was performed with MEGAX software (MEGA, RRID:SCR_000667) (Kumar et al., 2018). A maximum likelihood model (ML) with 1000 bootstrap replicates was applied to infer the phylogenetic tree. Via the Akaike information criterion implemented in MEGAX, the best-fit nucleotide substitution model was selected.

### Nucleotide Composition Analysis

Nucleotide composition analysis of HTNV complete coding sequences was analyzed using the CAIcal online server^6^ and CodonW local software^7^. Five critical compositional constraints were calculated. (I) The overall frequency of each nucleotide type (A, T/U, G, and C%). (II) Frequency of each nucleotide type at third synonymous codon position (A_3s_, U_3s_, G_3s_, and C_3s_%). (III) Mean frequencies of G + C nucleotides that occurred at first (GC_1s_), second (GC_2s_), and third (GC_3s_) synonymous codon position. (IV) The average of G + C nucleotides at first and second synonymous codon position (GC_12s_). (V) The overall GC and AU content. AUG and UGG, besides the three stop codons (UAA, UAG, and UGA), were excluded from the analysis: no synonymous codon.

### Relative Synonymous Codon Usage Analysis

The relative synonymous codon usage analysis (RSCU) value for a codon is “the observed frequency of that codon divided by the frequency expected under the assumption of equal usage of the synonymous codons for an amino acid” (Sharp and Li, 1987). The average RSCU values of HTNV overall genomes were calculated by the following formula as implemented in codonW software:

$$\begin{array}{c} RSCU=\frac{x_{ij}}{\sum_{j}^{n_{i}}x_{ij}}n_{i} \end{array}$$

Where *x*_*ij*_ is the number of occurrences of the *j*th codon for the *i*th amino acid encoded by *n*_*i*_ synonymous codons.

Relative synonymous codon usage analysis values greater than 1.0 refer to codons were more frequently used, and values less than 1.0 refer to codons were less frequently used, while value equal to 1.0 means that all codons were equally used (Sharp et al., 1986). Codons with RSCU values > 1.6 were considered over-represented, whereas codons with RSCU values < 0.6 were regarded as under-represented (Wong et al., 2010; Butt et al., 2014; Nasrullah et al., 2015; Rahman et al., 2018; Yao et al., 2020). The average RSCU values for host and natural reservoirs were obtained from the Codon and Codon-Pair Usage Tables database (CoCoPUTs).

### Effective Number of Codons Analysis

Effective number of codon (ENC) analysis was performed to estimate the degree of codon usage bias in HTNV coding sequences, independent of gene length and the number of amino acids. ENC was calculated using the following formula:

$$\begin{array}{c} ENC=2+\frac{9}{{\bar{F}}_{2}}+\frac{1}{{\bar{F}}_{3}}+\frac{5}{{\bar{F}}_{4}}+\frac{3}{{\bar{F}}_{6}} \end{array}$$

Where ${\bar{F}}_{k}$ (*k* = 2, 3, 4, and 6) is the mean of *F*_*k*_ values for the *k*-fold degenerate amino acids, which is calculated using the following formula:

$$\begin{array}{c} {\bar{F}}_{k}=\frac{n\sum_{i=1}^{k}{\left(\frac{n_{i}}{n}\right)}^{2}-1}{n-1} \end{array}$$

Where *n* is the total number of observations of the codons for that amino acid and *n*_*i*_ is the total number of events of the *i*th codon for that amino acid. ENC values can take values from 20 that indicate extreme codon usage bias using only one codon solely for each amino acid to 61 that show no preference using all possible synonymous codons equally (Wright, 1990; Comeron and Aguadé, 1998).

To elucidate whether HTNV coding sequences are constrained only by mutational pressure, ENC-plot was applied. HTNV genes for which the codon choice is only influenced by mutational bias will fall on or slightly under the expected ENC curve. Thus,

$$\begin{array}{c} ENC^{expected}=2+s+\left(\frac{29}{s^{2}+\left(1-s^{2}\right)}\right) \end{array}$$

Where *s* denotes GC_3s_ (Wright, 1990).

### Parity Rule 2 and Neutral Evolution Analyses

Parity Rule 2 (PR2) analysis and Neutral Evolution (Neutrality plot) analysis were performed to determine and compare the impact of mutation pressure and natural selection on the codon usage patterns of HTNV coding sequences.

**The PR2 analysis** “is a plot of AU-bias [A3/(A3 + U3)] as the ordinate and GC-bias [G3/(G3 + C3)] as the abscissa at the third codon position of the four-codon amino acids of entire genes. The center of the plot, where both coordinates are 0.5, is the place where A = U and G = C (PR2), with no bias between the influence of mutation pressure and natural selection on the codon usage of HTNV coding sequences” (Sueoka, 1995, 1999; Nasrullah et al., 2015).

**In the Neutrality plot**, the G + C content of the first and second synonymous codon positions (P_12_: ordinate) is plotted against the G + C content of the third synonymous codon position (P_3_: abscissa). A regression line was drawn between GC_1,2s_ and GC_3s_ values. “The regression coefficient against GC_3s_ is regarded as the mutation-selection equilibrium coefficient, and the evolutionary rates of the mutation pressure and natural selection pressure are expressed as the slopes of the regression line” (Sueoka, 1988; Nasrullah et al., 2015).

### Codon Adaptation Index

Codon adaptation index (CAI) is an effective measure of synonymous codon usage bias that predicts the gene expression level and assesses the adaptation of viral genes to their hosts. CAI values range from 0 to 1. Sequences with high CAI value shows high expression level and high adaptation to the host genome (Sharp and Li, 1987). The CAI values of HTNV coding sequences were calculated with the CAIcal online server using the synonymous codon usage patterns of *H. sapiens*, *A. agrarius*, and *N. confucianus* as references.

### Relative Codon Deoptimization Index

Relative codon deoptimization index (RCDI), a measure of codon deoptimization, was used to assess how similar the codon usage of a given gene is to the codon usage of a reference genome and test the deoptimization levels in viral genomes. An RCDI value close to one indicates the high similarity between viral and host genes. A low RCDI value indicates high adaptation to the host, while a high RCDI value indicates that some genes are expressed in the latency phase, or the virus might present a low replication rate (Mueller et al., 2006; Puigbò et al., 2010). The RCDI for HTNV coding sequences was calculated using RCDI/eRCDI online server^8^.

### Similarity Index

Similarity Index (SiD) is defined as the influence of the host’s overall codon usage on that of the virus. SiD was calculated as follow:

$$\begin{array}{c} R\left(A,B\right)=\frac{\sum_{i=1}^{59}a_{i}\times b_{i}}{\sqrt{\sum_{i=1}^{59}a_{i}^{2}\times \sum_{i=1}^{59}b_{i}^{2}}} \end{array}$$

$$\begin{array}{c} D\left(A,B\right)=\frac{1-R\left(A,B\right)}{2} \end{array}$$

*R(A, B)* represents a cosine value of an included angle between *A* and *B* particular vectors representing the degree of similarity between HTNV and a specific host at the aspect of the overall codon usage pattern, *a*_*i*_ is defined as the RSCU value for a particular codon in 59 synonymous codons of HTNV ORF, *b*_*i*_ is termed as the RSCU value for the same codon of the host. *D(A, B)* represents the potential effect of the host’s overall codon usage on that of HTNV, and this value ranges from zero to 1.0 (Zhou et al., 2013).

### Correspondence Analysis

Correspondence analysis (COA) is a multivariate statistical technique that assesses the relationship between multiple categorical variables. COA was used to investigate the major trends in codon usage variation within HTNV coding sequences (Greenacre, 1984). Each gene coding sequence was represented as a 59-dimensional vector (59 codons) and each dimension corresponded to the RSCU value of one codon (excluding AUG, UGG, and stop codons). COA based on the RSCU values was performed using the codonW program. R ggplot2 package (ggplot2, RRID:SCR_014601) (Wickham, 2011) was used to visualize all graphics presented in this study.

### Correlation Analysis

Correlation analysis, spearman’s method, was applied to investigate the relationship between compositional constraints, protein’s general average hydropathicity (GRAVY) and aromaticity (ARO), the first two principal axes of COA, and ENC in HTNV complete genomes. R corrplot package was used to perform All statistics (Wei and Simko, 2017). The codonW program obtained hydropathicity (GRAVY), aromaticity (ARO), and other related indicators of codon usage bias.

### Statistical Analyses

Mann–Whitney *U* test (abbreviated as Wilcox.test) and One-Way Analysis of Variance (ANOVA) test (abbreviated as ANOVA.test) were used to measure the significance with a threshold of *P* < 0.05 (McKnight and Najab, 2010; Kim, 2014; Qi et al., 2020; Yao et al., 2020).

## Results

### Recombination and Phylogenetic Analyses

Recombination events at either gene or genome-level can bias the inferred phylogenetic tree’s structure and the codon usage patterns (Schierup and Hein, 2000; Marais et al., 2001; Behura and Severson, 2013). Therefore, we checked all HTNV individual genomic segments for recombination events using the same method as reported before (Li et al., 2020). Out of 95 HTNV strains, seven strains were recombinants (Supplementary Table 1) and eliminated from further analysis. Whole-genome sequences of the remaining 88 strains were submitted to the phylogenetic analysis to investigate the codon usage patterns from an evolutionary perspective. A maximum likelihood method with GTR + G + I model and 1000 bootstrap replicates were applied to infer the phylogenetic tree. We found that HTNV strains evolved into three distinct clades (Supplementary Figure 1), named South Korean (SK, 67 isolates), Russian (RUS, four isolates), and Chinese (CH, 15 isolates). Two strains (X), AYW89-15 and Nc167, were considered out of the three clades due to their high genetic diversity. Thus, they were excluded from the clade-specific codon usage analysis.

### HTNV Genomes Are A- and U-Rich Composition

HTNV complete coding sequences’ compositional constraints were measured to evaluate their impact on the codon usage patterns. We found that the mean percentage of A (32.38 ± 0.23) and U (28.59 ± 0.15) were more frequent than G (21.81 ± 0.10) and C (17.22 ± 0.12) (Wilcox.test, *P* < 0.05). In the third synonymous codon position, A_3s_ (34.47 ± 0.69) and U_3s_ (34.40 ± 0.45) were also higher than G_3s_ (16.03 ± 0.32) and C_3s_ (16.02 ± 0.32) (Wilcox.test, *P* < 0.05). The mean percentage of AU and GC were (60.97 ± 0.17) and (39.03 ± 0.17), respectively, (Wilcox.test, *P* < 0.05), emphasizing that HTNV coding sequences are enriched with A and U nucleotides. The analysis of nucleotide content at first, second, and third synonymous codon position revealed that the mean frequency (%) of GC_1s_, GC_2s_, and GC_12s_ were (47.55 ± 0.15), (37.59 ± 0.12), and (42.57 ± 0.05), respectively, (Wilcox.test, *P* < 0.05). At the same time, the GC_3s_ and AU_3s_ were (31.13 ± 0.49) and (68.87 ± 0.49), respectively, (Wilcox.test, *P* < 0.05), showing that A/U nucleotides are preferred at the third codon position. These data confirmed that HTNV coding sequences are A- and U-rich composition (Supplementary Table 2).

### A- and U-Ended Codons Are Entirely Preferred in HTNV Genomes

**FIGURE 1:**
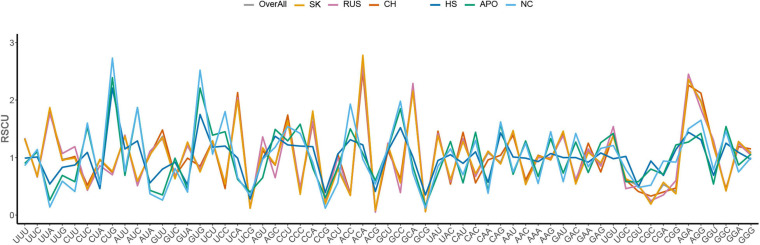
Comparative analysis of RSCU patterns between Hantaan virus (HTNV), clades, host, and natural reservoirs.

The RSCU analysis determines the codon usage patterns without the confounding influence of amino acid compositions. The average overall RSCU values of HTNV and its clades were calculated and compared with those of *H. sapiens*, *A. agrarius*, and *N. confucianus* (Figure 1 and Table 1). As expected from the nucleotide composition analysis, all of the 18 most abundantly used codons in HTNV genomes were A/U-ended, 10 (UUA, UCA, CCA, ACA, GCA, CAA, AAA, GAA, AGA, and GGA) were A-ended, and eight (UUU, AUU, GUU, UAU, CAU, AAU, GAU, and UGU) were U-ended (ANOVA.test, *P* < 0.05). Moreover, analysis of over and under-represented codons showed that eight of 59 codons (UUA [L], UCA [S], CCU [P], CCA [P], ACA [T], GCA [A], AGA [R], and AGG [R]) were over-represented (RSCU > 1.6) and A/U-ended except AGG [R]. All under-represented codons (17 codons, RSCU < 0.6) were G/C-ended except CGU [R] and CGA [R]. The rest 34 codons (RSCU values > 0.6 and < 1.6) were a combination between A/U-ended and G/C-ended (ANOVA.test, *P* < 0.05). The RSCU analysis confirmed that HTNV genomes exhibit an entire codon usage bias toward A/U-ended codons and comprise a relatively stable genetic composition at some specific levels. The analysis of the overall RSCU values within the three clades of HTNV showed that the codon usage preferences were the same, except for the Chinese clade that displayed a change in Pro [P], Gln [Q], and Gly [G] amino acids and Russian clade that exhibited a change only in Gly [G] amino acid. Although the 59 synonymous codon usages’ overall general trend within the three clades was relatively consistent, there was a statistically significant difference in their frequency (ANOVA.test, *P* < 0.05). These results suggested that the synonymous codon usage patterns play a role in the evolutionary processes of HTNV to some extent (Figure 1 and Table 1).

**TABLE 1:** Relative synonymous codon usage (RSCU) patterns of HTNV, host, and natural reservoirs.

Amino Acid	Codons	HTNV				Host and reservoirs		
		Overall	SK	RUS	CH	HS	APO	NC
Phe(F)	UUU	**1.33**	**1.33**	**1.34**	**1.32**	0.99	0.90	0.86
	UUC	0.67	0.67	0.66	0.68	**1.01**	**1.10**	**1.14**
Leu(L)	UUA	**1.85**	**1.87**	**1.76**	**1.81**	0.54	0.26	0.14
	UUG	0.97	0.96	1.07	0.95	0.83	0.69	0.59
	CUU	1.01	0.99	1.19	1.02	0.87	0.58	0.41
	CUC	0.46	0.45	0.43	0.52	1.09	1.53	1.60
	CUA	0.96	0.97	0.86	0.96	0.46	0.57	0.53
	CUG	0.75	0.76	0.70	0.74	**2.21**	**2.39**	**2.73**
Ile(I)	AUU	**1.37**	**1.37**	**1.39**	**1.37**	1.15	0.69	0.76
	AUC	0.58	0.59	0.51	0.59	**1.29**	**1.87**	**1.87**
	AUA	1.05	1.05	1.11	1.04	0.56	0.43	0.37
Val(V)	GUU	**1.37**	**1.35**	**1.33**	**1.48**	0.80	0.35	0.26
	GUC	0.65	0.63	0.67	0.68	0.92	0.99	0.81
	GUA	1.21	1.27	1.21	0.99	0.53	0.45	0.40
	GUG	0.77	0.75	0.80	0.85	**1.75**	**2.21**	**2.52**
Ser(S)	UCU	1.29	1.29	1.27	1.29	1.18	1.39	1.06
	UCC	0.52	0.54	0.56	0.46	1.20	1.45	**1.80**
	UCA	**2.03**	**2.00**	**2.04**	**2.13**	0.99	0.62	0.65
	UCG	0.12	0.12	0.12	0.13	0.28	0.40	0.34
	AGU	1.17	1.17	1.36	1.12	0.98	0.65	0.97
	AGC	0.87	0.88	0.65	0.87	**1.37**	**1.49**	1.18
Pro(P)	CCU	1.66	1.64	**1.63**	**1.74**	**1.22**	1.29	**1.54**
	CCC	0.38	0.36	0.48	0.44	1.20	**1.58**	1.42
	CCA	**1.76**	**1.81**	**1.63**	1.60	1.19	0.82	0.92
	CCG	0.20	0.19	0.26	0.22	0.39	0.30	0.12
Thr(T)	ACU	0.85	0.80	1.00	1.06	1.06	0.83	0.56
	ACC	0.35	0.34	0.34	0.37	**1.31**	**1.50**	**1.93**
	ACA	**2.72**	**2.78**	**2.61**	**2.50**	1.22	1.07	0.97
	ACG	0.08	0.08	0.05	0.07	0.41	0.60	0.54
Ala(A)	GCU	1.20	1.22	1.25	1.12	1.12	1.15	1.17
	GCC	0.57	0.56	0.39	0.63	**1.52**	**1.85**	**1.98**
	GCA	**2.17**	**2.16**	**2.29**	**2.19**	1.01	0.84	0.73
	GCG	0.06	0.06	0.07	0.06	0.35	0.16	0.12
Tyr(Y)	UAU	**1.40**	**1.38**	**1.42**	**1.46**	0.95	0.72	0.85
	UAC	0.60	0.62	0.58	0.54	**1.05**	**1.28**	**1.15**
His(H)	CAU	**1.31**	**1.28**	**1.33**	**1.44**	0.90	0.56	0.70
	CAC	0.69	0.72	0.67	0.56	**1.10**	**1.44**	**1.30**
Gln(Q)	CAA	**1.08**	**1.11**	**1.11**	0.96	0.57	0.42	0.38
	CAG	0.92	0.89	0.89	**1.04**	**1.43**	**1.58**	**1.62**
Asn(N)	AAU	**1.45**	**1.47**	**1.46**	**1.39**	**1.01**	0.71	0.75
	AAC	0.55	0.53	0.54	0.61	0.99	**1.29**	**1.25**
Lys(K)	AAA	**1.03**	**1.03**	1.00	**1.04**	0.93	0.67	0.55
	AAG	0.97	0.97	1.00	0.96	**1.07**	**1.33**	**1.45**
Asp(D)	GAU	**1.44**	**1.46**	**1.38**	**1.41**	1.00	0.73	0.58
	GAC	0.56	0.54	0.62	0.59	1.00	**1.27**	**1.42**
Glu(E)	GAA	**1.12**	**1.09**	**1.15**	**1.25**	0.92	0.74	0.84
	GAG	0.88	0.91	0.85	0.75	**1.08**	**1.26**	**1.16**
Cys(C)	UGU	**1.38**	**1.37**	**1.54**	**1.39**	0.98	**1.42**	**1.21**
	UGC	0.62	0.63	0.46	0.61	**1.02**	0.58	0.79
Arg(R)	CGU	0.51	0.53	0.51	0.41	0.49	0.58	0.48
	CGC	0.22	0.19	0.25	0.33	0.94	0.80	0.52
	CGA	0.53	0.57	0.35	0.40	0.69	0.71	0.94
	CGG	0.41	0.37	0.60	0.47	1.14	1.22	0.92
	AGA	**2.35**	**2.36**	**2.45**	**2.26**	**1.44**	1.27	1.50
	AGG	1.99	1.97	1.84	2.12	1.31	**1.42**	**1.65**
Gly(G)	GGU	1.23	1.23	**1.30**	**1.20**	0.69	0.54	0.79
	GGC	0.43	0.42	0.42	0.46	**1.25**	**1.54**	**1.46**
	GGA	**1.26**	**1.28**	1.24	1.19	1.09	0.87	0.75
	GGG	1.09	1.07	1.04	1.15	0.97	1.06	1.00

*Overall, overall genome; SK, South Korean clade; RUS, Russian clade; CH, Chinese clade; HS, *H. sapiens*; APO, *A. agrarius*; NC, *N. confucianus*. Bold represents preferred codons by HTNV, host, and natural reservoirs; Underlined codons denote the optimal codons of HTNV.*

**FIGURE 2:**
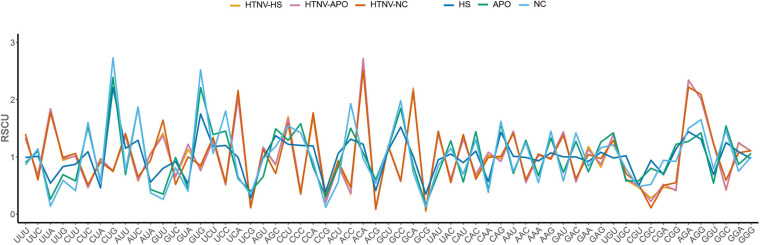
RSCU patterns of HTNV strains (based on isolation host) with its relevant host and natural reservoirs.

Regarding host and natural reservoirs’ RSCU analysis, HTNV codon usage patterns showed an inconsistency to those of its host and natural reservoirs, except for (AAU [N] and AGA [R]) concerning *H. sapiens* and (UGU [C]) regarding *A. agrarius* and *N. confucianus* (Figure 1 and Table 1). Furthermore, as our dataset comprises strains isolated from three different hosts, we performed an additional RSCU analysis based on the isolation host: where we calculated the average overall RSCU values of HTNV strains isolated from the three hosts (separately) and compared them with those of overall strains and their relevant hosts (Figure 2 and Supplementary Table 3). Compared with the codon usage patterns of overall strains, isolates of *A. agrarius* kept their patterns the same as overall strains. On the other hand, isolates of *H. sapiens* displayed a slight change where the virus used two codons with equal preference (CCU and CCA, RSCU = 1.70) for the translation of Pro [P] and (GGU and GGA, RSCU = 1.24) for Gly [G] amino acids: one like overall strains, and the other is different. Finally, isolates of *N. confucianus* showed a change in their codon usage preferences in two amino acids, Gln (Q) and Gly (G), while the rest preferred codons are the same as overall strains. Besides, there was a statistically significant difference in the frequency of all 59 synonymous codons between the isolates of the three hosts (ANOVA.test, *P* < 0.05). Concerning hosts, the antagonistic patterns we reported in overall strains, in general, still the same, but we noted a slight increase (one codon) in the preferred codons between the virus and *H. sapiens* to be three codons in total (CCU [P], AAU [N], and AGA [R]). Also, the same increase (one codon) was observed with *N. confucianus* to be two codons in total (CAG [Q] and UGU [C]), while with *A. agrarius*, there was only one commonly preferred codon (UGU [C]) with its HTNV isolates. These results might refer to the potential influence of the host and natural reservoirs in the codon bias choices in HTNV.

### Codon Usage Bias of HTNV Varies and Clade-Specific Among Genomic Segments

We performed the ENC analysis to assess the extent of codon usage bias among HTNV polyproteins and their relative clades (Supplementary Table 4). The mean ENC value for the overall genome in all HTNV strains was (47.52 ± 0.25) (Wilcox.test, *P* < 0.05), revealing that the codon usage bias among different HTNV strains was relatively stable and similar. Moreover, the mean ENC values in the isolates of each clade, whole-genome level, were 47.46 ± 0.18, 47.36 ± 0.12, and 47.73 ± 0.2 regarding South Korean, Russian, and Chinese clades, respectively, (Wilcox.test, *P* < 0.05). Concerning HTNV individual genomic segments ENC analysis, clades-specific, Chinese clade showed a specific difference from South Korean and Russian clades. S-segment of Chinese clade showed the highest ENC mean value (51.09 ± 0.66) compared with South Korean (49.64 ± 0.63) and Russian (49.04 ± 0.50) clades (Wilcox.test, *P* < 0.05). Regarding L-segment, we found the same situation; the mean ENC value of Chinese clade was the highest (47.02 ± 0.47), followed by South Korean (45.69 ± 0.31) and Russian (45.16 ± 0.26) clades (Wilcox.test, *P* < 0.05). On the contrary, the mean ENC values for M-segment of South Korean and Russian clades were approximately the same (49.52 ± 0.51 and 49.51 ± 0.25) (Wilcox.test, *P* > 0.05) and higher than that of Chinese clade (47.44 ± 0.38) (Wilcox.test, *P* < 0.05). Altogether, HTNV genomes showed a stable average ENC value (47.52 > 35), indicating lower codon usage bias, thus a relatively conserved genomic composition.

### Mutational Bias Effect on Codon Usage Patterns of HTNV

**FIGURE 3:**
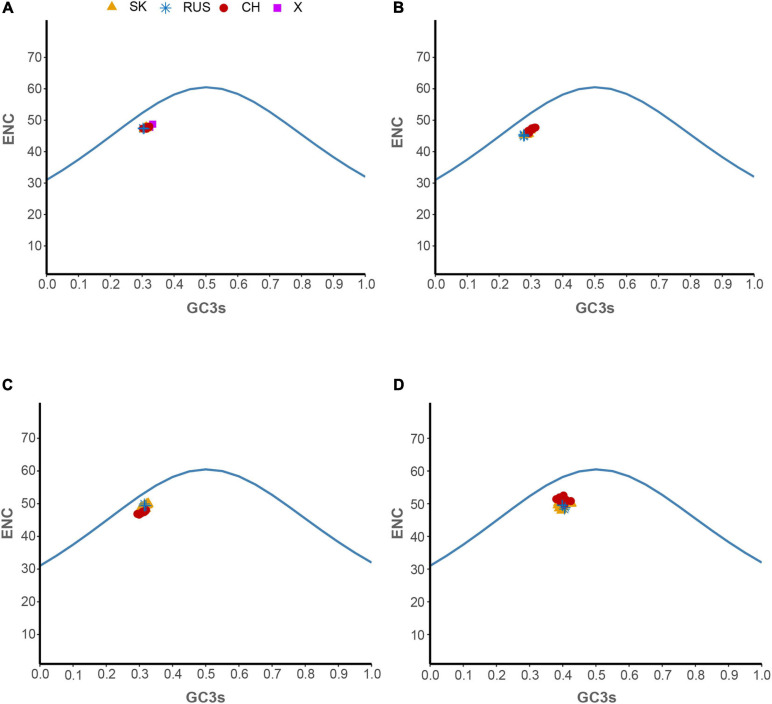
ENC-plot analysis. **(A)** Overall genome, **(B)** L-segment, **(C)** M-segment, and **(D)** S-segment. The continuous blue curve represents the expected ENC if GC3_s_ composition solely constrains the codon usage bias. X represents isolates were regarded as out of three phylogenetic clades. The color coding is the same in all plots.

**FIGURE 4:**
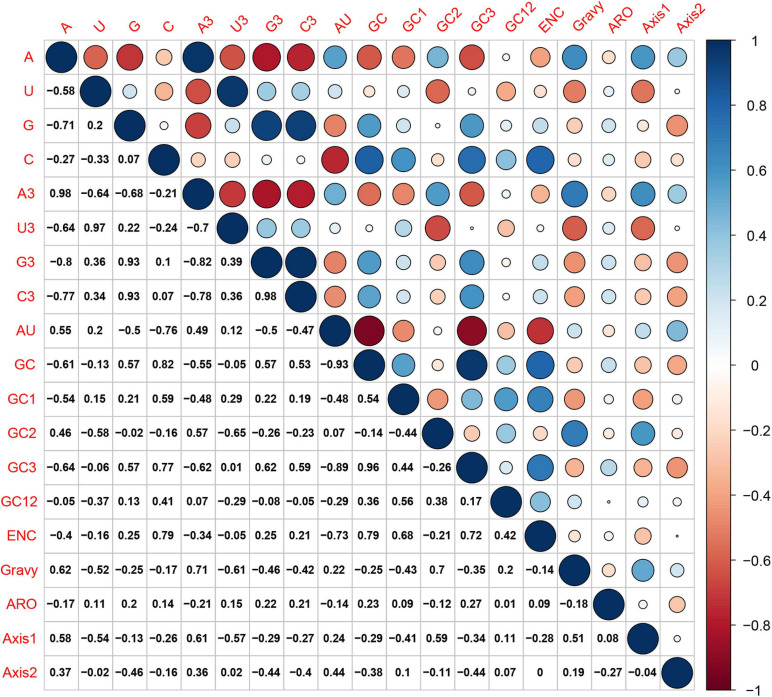
Spearman’s correlation analysis between compositional constraints, ENC, Gravy, ARO, and the first two principal axes of COA in HTNV whole genome. Dark blue, positive correlation; Dark red, negative correlation. The high color darkness means a highly significant correlation and vice-versa.

Hantaan virus genomes showed a strong bias toward A and U compositional constraints and a complete preference toward A- and U-ended codons in their codon usage patterns. These results broadly refer to the presence of mutation pressure effect in shaping the codon usage patterns of HTNV. To further investigate the effect of mutation pressure on the patterns of HTNV codon usage, we constructed the ENC plot based on the overall genome and each genomic segment according to the phylogenetic clades. We found that all HTNV genes assembled below the expected ENC curve (Figure 3). This result indicated that other factors, including natural selection, influence the evolution of HTNV codon usage along with mutation pressure. Moreover, we noted a combination of significant positive and negative correlations between the compositional constraints, ENC, and the first two principal axes of COA (Figure 4). All the above results confirm the mutation pressure influence in shaping the codon usage patterns in HTNV genomes.

### Natural Selection Effect on Codon Usage Patterns of HTNV

**FIGURE 5:**
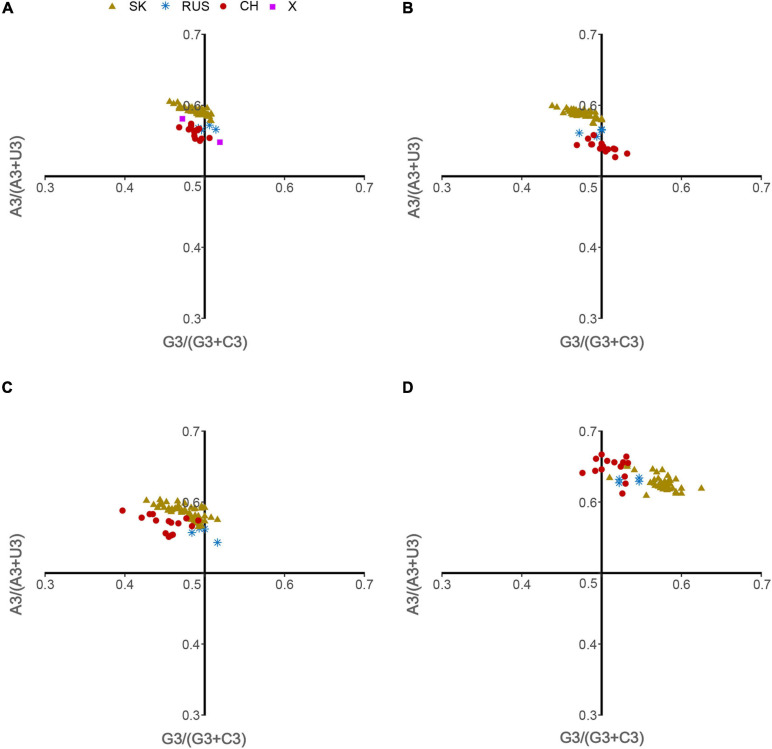
Parity Rule 2 (PR2) analysis. **(A)** Overall genome, **(B)** L-segment, **(C)** M-segment, and **(D)** S-segment.

**FIGURE 6:**
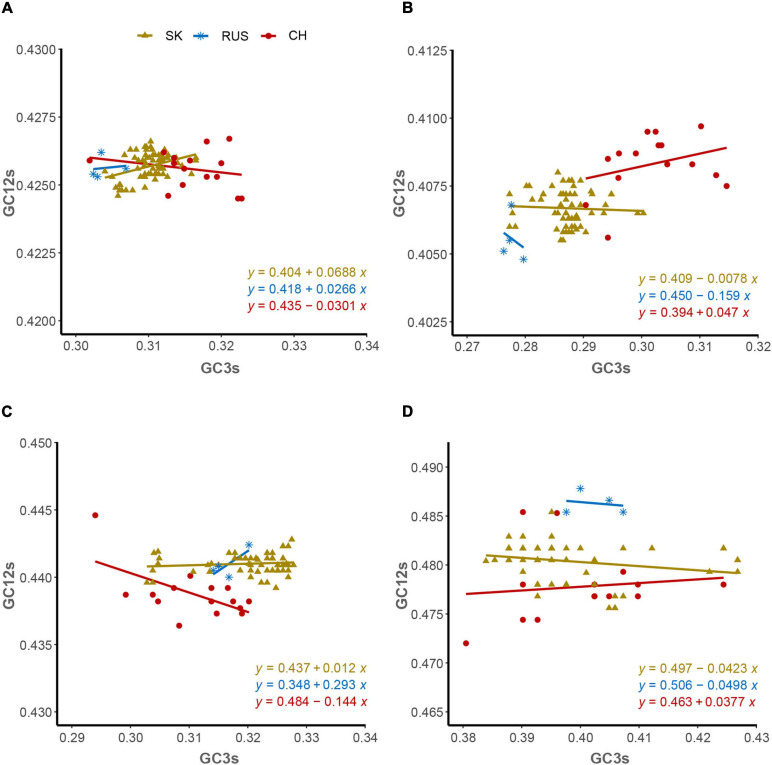
Neutrality plot analysis. **(A)** Overall genome, **(B)** L-segment, **(C)** M-segment, and **(D)** S-segment.

The ENC-plot analysis showed that apart from mutational bias, other factors, such as natural selection, have the main role in shaping the codon usage bias of HTNV coding sequences. To further determine and compare the effect of natural selection and mutation pressure on the codon usage preferences, we performed a PR2 analysis. In PR2 analysis, the relation between A–U content and G–C content in the four-fold degenerate codon families (Ala [A], Arg [R], Gly [G], Leu [L], Pro [P], Ser [S], Thr [T], and Val [V]) was determined to examine whether the biased codon choices are restricted in highly biased protein-coding genes. We observed a remarkable bias toward A–U over G–C in the four-fold degenerate codon families (Figure 5). Although this result confirms the presence of both mutation pressure and natural selection, it reflects the dominant influence of natural selection on shaping the patterns of codon usage bias in HTNV. Moreover, we constructed the neutrality plot that detects the extent of variation between mutation pressure and natural selection on shaping the codon usage bias. For HTNV overall genome, a significant positive correlation (*r* = 0.2, *P* = 0.07) was found between GC12_s_ and GC3_s_ with correlation coefficient of 0.0213 ± 0.419 (Supplementary Figure 2). Therefore, the degree of mutation pressure effect was calculated to be 2.13%, whereas the natural selection was calculated to be 97.87%, revealing the dominant effect of natural selection on the patterns of HTNV codon usage. Although the clade-specific analysis results were slightly different in mutation pressure and natural selection’ magnitude from the overall genome analysis, natural selection still exerts the dominant effect on the codon bias choices in HTNV (Figure 6A). In the South Korean clade, a significant positive correlation (*r* = 0.37, *P* = 0.002) was obtained with a regression line slope of 0.0688, so the effect of mutation pressure was calculated to be 6.88%, while the natural selection to be 93.12%. A non-significant negative correlation (*r* = −0.23, *P* > 0.05) was found between GC12s and GC3s with a regression line slope of 0.0301, so the effect of mutation pressure was found in the Chinese clade calculated to be 3.01%, while the natural selection to be 96.99%. In the Russian clade, the correlation between GC12_s_ and GC3_s_ was also non-significant, *P* > 0.05 with a regression line slope of 0.0266, indicating the prevailing leverage of natural selection in shaping HTNV codon usage patterns (Figure 6A). Additionally, a Neutrality plot analysis, clade-specific, on each genomic segment was performed. Compared to the strong influence of natural selection observed in the overall genome level, the effect of mutation pressure showed a relative increase at the genomic segment level. The effect of mutation pressure was relatively higher on L, M, and S segments in the isolates of the Russian clade with a slope of 0.159, 0.293, and 0.0498, respectively. In the isolates of the Chinese clade, the slopes of regression lines in L, M, and S segments were 0.047, 0.144, and 0.0377, respectively, while, in the isolates of the South Korean clade, the effect of mutation pressure was relatively low on L, M, and S segments with slopes of 0.0078, 0.0017, and 0.0423, respectively, (Figures 6B–D). According to these results, despite the variation of mutation pressure effect in HTNV individual genomic segments, natural selection still predominates the codon bias choices. Furthermore, we found a mixture of significant and non-significant correlations between the general average hydropathicity (GRAVY), aromaticity (ARO), the first two principal axes of COA, ENC, and several compositional constraints (Figure 4). The natural selection effect on codon usage bias was further confirmed by comparing the preferred codons between HTNV, host, and natural reservoirs through three analyses CAI, RCDI, and SiD, as shown in the coming sections.

### Trends of Codon Usage Variation in HTNV

**FIGURE 7:**
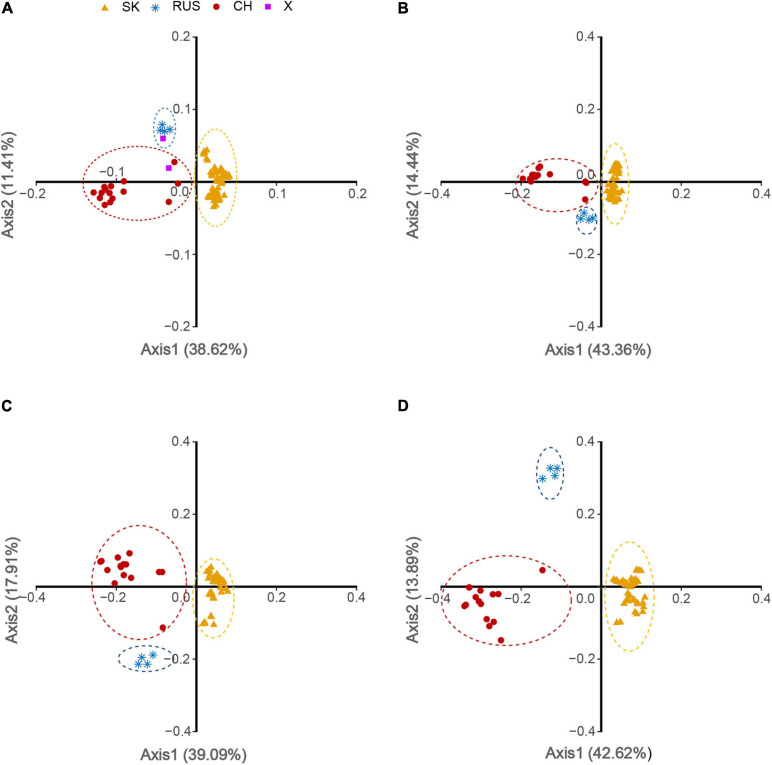
Correspondence analysis (COA) of HTNV based on RSCU values. **(A)** Overall genome, **(B)** L-segment, **(C)** M-segment, and **(D)** S-segment.

To examine the variations in the synonymous codon usage among the coding sequences of HTNV strains, we performed a COA on the overall genome and each genomic segment (L, M, and S) individually based on the RSCU values. Although COA creates a series of dimensions to identify trends that explain the data variation, the first two dimensions account for most data inertia (Greenacre, 1984). We used the values of these two axes to draw the COA plots, where each strain represented by a point, and the distance between strains give a degree of similarity or dissimilarity in the codon usage patterns (Figure 7). The first (f′1) and second (f′2) principle axes that account for majority of data inertia were Overall: f′1 = 38.62%, f′2 = 11.41% (Figure 7A); L-segment: f′1 = 43.36%, f′2 = 14.44% (Figure 7B); M-segment: f′1 = 39.09%, f′2 = 17.91% (Figure 7C); S segment: f′1 = 42.62%, f′2 = 13.89% (Figure 7D). The distribution of HTNV strains on the axes plots showed that three separate clusters assembled at different places. Cluster A consisted of 67 isolates, Cluster B consisted of five isolates, and Cluster C consisted of 16 isolates (Figure 7A). The clustering was clearer at the genomic segment level, especially for the S segment (Figures 7B–D). Interestingly, we found that HTNV strains clustered appropriately with the clusters obtained from the phylogenetic analysis. Isolates of the South Korean clade (67 isolates) formed the largest cluster (given dark yellow color in the plots). The Russian clade (4 isolates) formed the second cluster (given steel blue color in the plots). The Chinese clade (15 isolates) formed the third cluster (given dark red color in the plots). The two strains (AYW89-15 and Nc167) reported as out of the three phylogenetic clades, one found to cluster with the Russian clade and the second with the Chinese clade (given purple color in the plot) (Figure 7A). Another interesting observation, when looking at the distribution of HTNV strains from a regional basis, we found that some strains isolated from China are circulating in clusters A and B, which mainly consisted of strains isolated from South Korea, and Russia, respectively. This result suggested that the common ancestor of HTNV clades might originate in China.

### HTNV Codon Usage Displays the Highest Adaptation to *H. sapiens*

**FIGURE 8:**
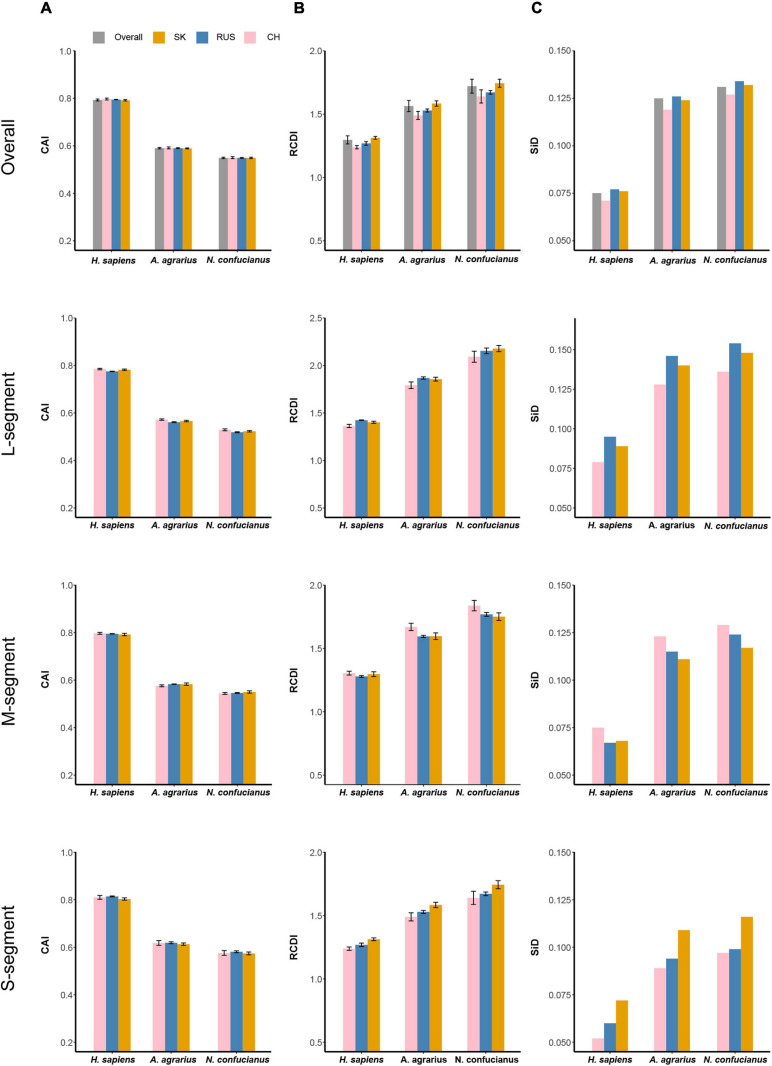
Codon usage adaptation indices of HTNV to its host and natural reservoirs. **(A)** Codon adaptation index (CAI), **(B)** relative codon deoptimization index (RCDI), and **(C)** similarity index (SiD). Standard deviation was showed in **(A)** and **(B)**.

Codon adaptation index analysis was performed to measure the codon usage optimization and adaptation of HTNV to its host and natural reservoirs. Concerning *H. sapiens*, *A. agrarius*, and *N. confucianus*, the mean CAI values of HTNV overall genomes were 0.793 ± 0.004, 0.588 ± 0.003, and 0.549 ± 0.003, respectively, indicating higher codon usage adaptation and expression level to *H. sapiens* than *A. agrarius* and *N. confucianus* (Wilcox.test, *P* < 0.05). The CAI values of each genomic segment were also calculated for each phylogenetic clade concerning *H. sapiens*, *A. agrarius*, and *N. confucianus*. The values obtained for the three HTNV clades were relatively similar; however, the highest values were for the S segment in the isolates of Russian clade (0.814 ± 0.002) to *H. sapiens*, (0.619 ± 0.004) to *A. agrarius*, and (0.581 ± 0.004) to *N. confucianus* (Wilcox.test, *P* > 0.05). M segment comes next after the S segment with the highest value in the Chinese clade (0.797 ± 0.004) to *H. sapiens* (Wilcox.test, *P* < 0.05), whereas the values in the Russian and South Korean were the same (0.583) to *A. agrarius* (Wilcox.test, *P* > 0.05), and the highest value was in the South Korean clade (0.550 ± 0.005) to *N. confucianus* (Wilcox.test, *P* < 0.05). L segment obtained the highest CAI values also in the isolates of Chinese clade (0.785 ± 0.003) to *H. sapiens*, (0.572 ± 0.003) to *A. agrarius*, and (0.529 ± 0.004) to *N. confucianus* (Wilcox.test, *P* < 0.05) (Figure 8A and Supplementary Table 5). From these results, HTNV codon usage adaptation and expression level are the highest in *H. sapiens* compared with *A. agrarius* and *N. confucianus*, while S segment is the highest part of HTNV genome adapted to the host and natural reservoirs.

### HTNV Shows the Highest Codon Deoptimization to *N. confucianus*

Relative codon deoptimization index analysis was applied to compare the similarity in codon usage of HTNV coding sequences to its host and natural reservoirs. Mean RCDI values of HTNV overall genomes were 1.882 ± 0.024, 1.672 ± 0.019, 1.33 ± 0.017, concerning *N. confucianus*, *A. agrarius*, and *H. sapiens*, respectively, (Wilcox.test, *P* < 0.05). Furthermore, clade-specific RCDI values of each genomic segment were calculated. The highest RCDI values were for the L segment in the isolates of the South Korean clade (2.178 ± 0.033) to *N. confucianus*, while isolates of the Russian clade showed the highest values (1.868 ± 0.012) to *A. agrarius*, and (1.424 ± 0.003) to *H. sapiens* (Wilcox.test, *P* < 0.05). M segment comes after the L segment with the highest values in the isolates of Chinese clade (1.838 ± 0.041) to *N. confucianus*, (1.670 ± 0.029) to *A. agrarius*, and (1.305 ± 0.015) to *H. sapiens* (Wilcox.test, *P* < 0.05). S segment obtained the lowest RCDI values compared with L and M segments, with the highest values in the isolates of South Korean clade (1.744 ± 0.023) to *N. confucianus*, (1.584 ± 0.021) to *A. agrarius*, and (1.313 ± 0.011) to *H. sapiens* (Wilcox.test, *P* < 0.05) (Figure 8B and Supplementary Table 6). These results showed that the codon deoptimization in HTNV is the highest for *N. confucianus*, followed by *A. agrarius*, and the lowest for *H. sapiens*. HTNV codon deoptimization is clade-specific and varies among genes, and L segment displayed the highest codon deoptimization for host and natural reservoirs.

### Selection Pressure by *N. confucianus* Is the Strongest on HTNV

Similarity Index analysis was performed to evaluate the potential role of the overall codon usage pattern of *H. sapiens*, *A. agrarius*, and *N. confucianus* on the formation and evolution of the overall codon usage in HTNV. The index value of HTNV polyproteins was found to be the highest for (*N. confucianus vs.* HTNV) group followed by (*A. agrarius vs.* HTNV), and the lowest was for (*H. sapiens vs.* HTNV), indicating that the effect of *N. confucianus* and *A. agrarius* in the formation of the overall codon usage of HTNV is relatively stronger than *H. sapiens*. Clade-wise SiD values for each genomic segment were also computed to host and natural reservoirs. We found that the effect of host and natural reservoirs on each genomic segment was relatively the same; *N. confucianus* displayed the highest effect on shaping HTNV codon usage patterns, followed by *A. agrarius*, then *H. sapiens*. However, the L segment effect was the highest compared with M segment and S segment. Furthermore, we found that the S segment SiD’s highest values were in the South Korean clade, while, M segment’s highest values were in the Chinese clade and the L segment’s highest values were in the Russian clade (Figure 8C). Altogether, selection pressure by *N. confucianus* in the formation and evolution of HTNV codon usage is higher than *A. agrarius* and *H. sapiens*. Among HTNV genomic segments, the codon usage evolution of L segment is highly induced by the host and natural reservoirs compared with M and S segments.

## Discussion

Our report presents a comprehensive analysis of the codon usage bias and various factors shaping its patterns in HTNV strains isolated from China, Russia, and South Korea. We followed the same strategy (phylogenetic-dependent codon usage analysis) as reported before (Butt et al., 2016; Qi et al., 2020).

### Codon Usage Patterns Relatively Restrict the Evolution of HTNV

The inferred phylogenetic tree based on whole-genome showed that HTNV strains enrolled in our study divided into three distinct clades. We named them, based on a regional basis, South Korean, Russian, and Chinese. Our result was consistent with a recently reported phylogenetic analysis of HTNV (Li et al., 2020). Moreover, the investigation of the codon usage variation major trends in HTNV genomes via COA, based on RSCU values, revealed that HTNV strains also assembled into three clusters consistently with the phylogenetic clades, suggesting that the codon usage patterns might play a role in the evolution of HTNV. Furthermore, we observed some strains isolated from China are circulating in the South Korean (e.g., JS10) and the Russian (e.g., Fuyuan-Aa-26) clades. Although our observation cannot determine the origin or dissemination of the virus, it supports, to some extent, the hypothesis of Li et al. that the common ancestor of HTNV probably first emerged in China (Li et al., 2020).

### Compositional Constraints Influence the Codon Usage Patterns of HTNV

Genome’s overall nucleotide content can largely affect the codon usage patterns (Jenkins and Holmes, 2003). Additionally, van Hemert et al. (2016) suggested that the nucleotide bias in RNA virus genomes is the primary determinant of the specific codon usage, limiting the role for codon selection and translational control. Therefore, we carefully analyzed the nucleotide composition in HTNV genomes. The analysis revealed that HTNV genomes are A- and U-rich composition, similar to other RNA viruses (Rahman et al., 2018; Khandia et al., 2019; Tort et al., 2020). An RSCU analysis showed that, as expected from nucleotide composition analysis, HTNV genomes entirely prefer A- and U-ended codons, indicating the potential role of compositional constraints (mutational bias) in shaping the codon usage patterns of HTNV. Clade-specific RSCU analysis showed that the 59 synonymous codon usage’s overall general trend in the isolates of the three clades was comparatively consistent, supporting the assumption that the codon usage patterns restrict the evolution of HTNV to some extent.

### Codon Usage in HTNV Coding Sequences Is Slightly Biased

The remarkable bias toward A and U nucleotides composition with a significant preference for A- and U-ended codons in HTNV coding sequences urged us to determine the overall codon usage bias in HTNV genomes via ENC analysis. An ENC value of a gene is inversely related to the expression level of that gene (Wright, 1990). In general, an ENC value > 35 suggests that there is a relatively stable and conserved genomic composition (Comeron and Aguadé, 1998; Kim et al., 2020). The analysis showed that HTNV overall genomes have a mean value of 47.52, indicating a slightly biased and relatively stable codon usage in HTNV genomes. Our result was consistent with both negative and positive ssRNA viruses that displayed a low codon usage bias. Negative ssRNA viruses include EBOV (ENC, 55.57), MARV (ENC, 54.2), CCHFV (ENC, 52.34), RABV (ENC, 53.84), and RVFV (Nasrullah et al., 2015; Rahman et al., 2018; Zhang et al., 2018; Kim et al., 2020; Luo et al., 2020). Positive ssRNA viruses include HCV (ENC, 52.62), DENV (ENC, 49.70), CHIKV (ENC, 55.56), ZIKV (ENC, 53.21), and SARS-CoV-2 (ENC, 48.54) (Hu et al., 2011; Ma et al., 2013; Butt et al., 2014; Tao and Yao, 2020; Tort et al., 2020). Previous studies suggested that the diversity of codons encoding amino acids (low codon usage bias) and low gene expression in RNA viruses might minimize the translation machinery competition between the virus and the host that maximize its replication rate within the host genome (Jenkins and Holmes, 2003; Butt et al., 2014; Nasrullah et al., 2015). Clade-specific ENC analysis showed relatively similar values within and between HTNV different genomes in the isolates of the three clades. Our result was consistent with the data obtained from the analysis of over-and under-represented RSCU, suggesting that HTNV comprises a relatively stable genetic composition at some specific levels. Notably, we found that the degree of codon usage bias varies among the three genomic segments and clade-specific. S segment showed the lowest codon bias with an ENC value of 51.09 in the isolates of the Chinese clade, indicating that the S segment uses a greater variety of codons than other segments.

### Natural Selection Predominates the Evolution of HTNV Codon Usage

The primary factors that account for codon usage variation among genes in different organisms are natural selection and mutational pressure coupled with genetic drift (Bulmer, 1991; Labella et al., 2019; Tort et al., 2020). Nucleotide composition and RSCU analyses revealed the mutation pressure’s potential role in shaping the codon usage bias of HTNV; nevertheless, we still need to investigate whether mutation pressure solely shapes the codon usage patterns or other factors, including natural selection, are involved in the evolution of HTNV. Besides, if both mutation pressure and natural selection contribute to the selection of biased codon choices, what is the extent of each factor? Which factor has the dominant effect on the selection of biased codon choices? To answer these questions, we conducted a series of analyses. The ENC-plot analysis showed that all HTNV genes assembled under the expected ENC curve, elucidating the contribution of both mutational bias and translational selection on selecting the biased codon choices. If mutation pressure solely constrains shaping the codon usage patterns, the occurrence of nucleotides A and U should be equal to that of C and G at the third synonymous codon position (Zhang et al., 2013; Butt et al., 2014). The PR2 analysis showed that A and U nucleotides were more frequent than G and C at the third codon position in the fourfold degenerate codon families. This result referred to the significant role of natural selection in shaping the codon usage patterns in HTNV genomes. Moreover, Neutrality plot analysis showed the dominant effect of natural selection on the codon usage patterns in overall genomes and each genomic segment in the isolates of HTNV clades. Finally, we finished our investigation with the most potent analysis, CAI analysis, to explore the effect of natural selection on biased codon choices. The CAI value of a gene is positively related to the expression level and the translational selection’s effect on shaping that gene’s codon usage bias (Sharp and Li, 1987; Carbone et al., 2003). The analysis confirmed the prevalent leverage of natural selection on shaping the codon usage patterns in HTNV, as discussed in the coming section.

### HTNV Codon Usage Evolution Is Host and Natural Reservoir Specific

CAI analysis is frequently used to evaluate the codon usage optimization, gene expression, and adaptation of viral genes to their hosts (Rahman et al., 2018; Khandia et al., 2019; Tort et al., 2020). The CAI analysis revealed the high adaptation of HTNV whole-genomes to *H. Sapiens* with a value of 0.793 compared with its natural reservoirs, *A. agrarius* with a value of 0.588, and *N. confucianus* with a value of 0.549. The analysis revealed that natural selection from both host and natural reservoirs had influenced the codon usage patterns of HTNV. The high adaptation of HTNV to *H. sapiens* reflected the adjustment of HTNV codon usage patterns to the best fit of those of *H. sapiens* to achieve the highest replication rate that interprets the high pathogenicity of HTNV in humans (Avšič-Županc et al., 2019). The slightly low adaptation of HTNV to its natural reservoirs indicated that HTNV had maintained a low surviving translation rate of its proteins within its natural reservoirs without causing any disease symptoms; however, it cannot negate the harm of the virus on them too (Meyer and Schmaljohn, 2000). Clade-specific CAI analysis for each genomic segment showed that S segment is the highest virulence part in HTNV genome as it obtained the highest adaptation value to host and natural reservoirs concerning the Russian clade. Whereas, L segment showed the lowest pathogenicity toward host and reservoirs, regarding Russian clade, as it acquired the lowest adaptation value and gene expression level to the host and natural reservoirs.

In contrast to CAI, RCDI analysis measures the codon deoptimization levels in viral genomes relative to the host genome’s codon usage (Khandia et al., 2019; Dutta et al., 2020; Luo et al., 2020). The results of RCDI analysis for HTNV whole-genomes are consistent and confirmed the results of CAI. HTNV genomes showed the highest codon deoptimization level in *N. confucianus*, followed by *A. agrarius*, confirming that the similarity of codon usage patterns between HTNV and those of *N. confucianus* and *A. agrarius* was not so high enough to allow efficient expression of viral genes. Moreover, the viral genes might express in the latency phase within the genomes of its natural reservoirs. Concerning *H. sapiens*, the RCDI was the lowest, indicating the high similarity and adaptation of codon usage patterns between HTNV and *H. sapiens*, giving the virus the ability to express its genes efficiently with a high replication rate.

Viruses, as parasitic organisms, select their optimal codons depending mainly upon their hosts (Butt et al., 2016). Previous studies suggested that the evolution of viruses takes three patterns of codon usage compared with their hosts. Coincident patterns allow the corresponding amino acids to be translated efficiently within the host’s genome, such as poliovirus (Mueller et al., 2006). Antagonistic patterns allow viral proteins to be folded adequately, such as hepatitis A virus (Sánchez et al., 2003). A mixture of coincident and antagonistic patterns gives the virus the advantage to adapt to several hosts, vectors, and environments such as ZIKV (Butt et al., 2016). The comparison of HTNV codon usage preferences with those of its host and natural reservoirs revealed that HTNV had evolved relatively antagonistic codon usage patterns. Regarding *N. confucianus* and *A. agrarius*, this result might be acceptable as the virus showed low adaptation and high codon deoptimization levels, but the situation in *H. sapiens* was different. Although the virus displayed high adaptation and low codon deoptimization levels, it showed partial antagonistic codon usage patterns. Zhao et al. (2003) gave a possible explanation for this phenomenon in A/U-rich viruses. We had only compared the average codon usage between HTNV genes and human genes. Moreover, humans’ codon preferences comprise vast differences among their genes (Ikemura, 1985). Therefore, HTNV genes might have some similarities in codon choice to some local human genes that provide HTNV genes with a selective advantage for translation and replication in specific locations in the human body (Zhao et al., 2003). This explanation might interpret the increased pathogenicity of HTNV, due to high viral production, in particular parts of the human body, causing a specific clinical syndrome (Krautkrämer et al., 2013).

Estimating the synonymous codon usage effect of the hosts on that of specific viruses by comparing their individual RSCU values obscure our awareness about the hosts’ overall codon usage effect on the formation and evolution of that of the viruses (Butt et al., 2014). Therefore, we performed the SiD analysis. We found that selection pressure from the host and natural reservoirs had contributed to the evolution of HTNV codon usage. *N. confucianus* showed the strongest effect on the formation of the overall codon usage of HTNV compared with *A. agrarius* and *H. sapiens*. This finding might interpret the isolation of the novel HTNV strain (Nc167) from *N. confucianus* in China: displayed a higher genetic diversity than all other strains enrolled in our study (Wang et al., 2000). Nonetheless, the selection pressure displayed by *A. agrarius* on the formation and evolution of overall HTNV codon usage still predominant over *N. confucianus* and *H. sapiens*, as out of 95 strains enrolled in our study, we observed that 54 strains were isolated from *A. agrarius* (the ancient and primary natural reservoir of HTNV). These results might indicate a stable, remarkable preference and interaction between HTNV and *A. agrarius* as a natural reservoir that affects the overall virus survival, spread, adaptation, and evolution. The RSCU analysis based on the isolation host showed that the main differences in the codon usage preferences between the strains of three hosts were in three amino acids Pro [P], Gln [Q], and Gly [G]. Interestingly, the differences in the codon usage preferences displayed by Chinese and Russian Clades were also in the same amino acids Pro [P], Gln [Q], and Gly [G], confirming the role of the host and natural reservoirs in shaping the codon bias choices in HTNV and support our hypothesis that the virus had evolved host and natural reservoir specific.

The dinucleotide composition role in shaping the codon usage patterns in different hantaviruses segments was previously reported (Sankar et al., 2015). In this study, the authors proposed that the mutation pressure is the main factor shaping hantaviruses codon usage patterns: in detail, the evolution of M and S segments are mainly subjected to mutation pressure and L segment to natural selection. In our study, like their findings, we reported the effect of both mutation pressure and natural selection and noted an increase in the mutation pressure magnitude in M and S segments in the isolates of Russian and Chinese clades. However, unlike their assumption, we found that natural selection is the dominant factor in shaping HTNV codon bias choices in all three segments. We return this discrepancy to definite limitations in Sanker et al. study. They enrolled an unequal number of sequences (for each segment, *L* = 23, *M* = 43, *S* = 51) from different hantaviruses species and analyzed them together that certainly obscured each species’ evolutionary processes and led to misleading overall findings. Analyzing the codon usage patterns to elucidate the evolutionary pressures in a virus genome require a sufficient number of strains with full genomic sequences for that virus. Moreover, they depended mainly on the dinucleotide composition analysis to derive their hypothesis, ignoring the virus-host interaction aspect. In contrast, we analyzed the codon usage patterns in a specific genotype (HTNV) with many strains and complete genomic sequences linked with specific hosts via a plethora of codon usage indices. Additionally, we explored the virus-host adaptability via four different codon adaptation indices: RSCU, CAI, RCDI, and SiD.

The principal remaining obstacle that limits our understanding of hantaviruses’ evolutionary processes is the lack of full genomic sequences, particularly those isolated from the host and natural reservoirs. Thus, further scientific research should be executed in this field in the future. Moreover, future experimental research must be conducted on HTNV to establish viral adaptation in various aspects and hosts.

## Conclusion

Both mutation pressure and natural selection impact the codon usage patterns of HTNV; however, natural selection is the dominant factor influencing its codon usage bias. Host and natural reservoirs played a significant role in the selection of biased codons choices and the evolution of HTNV. No remarkable difference was found at the overall genome level between the isolates of three HTNV clades regarding the codon usage patterns and the molecular evolutionary processes. In contrast, the evolution of the individual genomic segments was clade-specific as we observed a variation between the isolates of three HTNV clades in the degree of codon usage bias and the codon adaptation indices to host and natural reservoirs. Among HTNV’s three genomic segments, S segment displayed codon usage patterns, bias, adaptation, and evolutionary processes, introduced it as the most pathogenic part of HTNV genome. Finally, this is the first comprehensive study on codon usage bias and various factors shaping its patterns in HTNV genomes to the best of our awareness. Our study will help better understand the evolutionary dynamics of HTNV that will serve its future basic research and aid live attenuated vaccines design. Moreover, our study opens the door for a more genotype-specific codon usage analysis for all hantaviruses species to reveal their overall molecular evolutionary dynamics and adaptability to several hosts and environments.

## Data Availability Statement

The datasets presented in this study can be found in online repositories. The names of the repository/repositories and accession number(s) can be found in the article/Supplementary Material.

## Author Contributions

ST conceived and designed the experiments. GA, HW, and HB performed all experiments. GA and XY collected and analyzed the data. GA drafted the manuscript. All authors read and approved the final manuscript.

## Conflict of Interest

The authors declare that the research was conducted in the absence of any commercial or financial relationships that could be construed as a potential conflict of interest.

## Footnotes

1 https://talk.ictvonline.org/taxonomy/

2 https://www.viprbrc.org

3 http://www.ncbi.nlm.nih.gov/GenBank

4 https://www.bioinformatics.nl/cgi-bin/emboss/supermatcher

5 https://hive.biochemistry.gwu.edu/review/codon2

6 http://genomes.urv.es/CAIcal/

7 http://sourceforge.net/projects/codonw

8 http://genomes.urv.es/CAIcal/RCDI/
