# Supplementary material for: Edging on Mutational Bias, Induced Natural Selection From Host and Natural Reservoirs Predominates Codon Usage Evolution in Hantaan Virus
Source: Front Microbiol. 2021 Jul 2;12:699788. doi: 10.3389/fmicb.2021.699788 (PMC8283416; doi:10.3389/fmicb.2021.699788)
Supplement: Supplementary file 1 [file Data_Sheet_1.PDF]

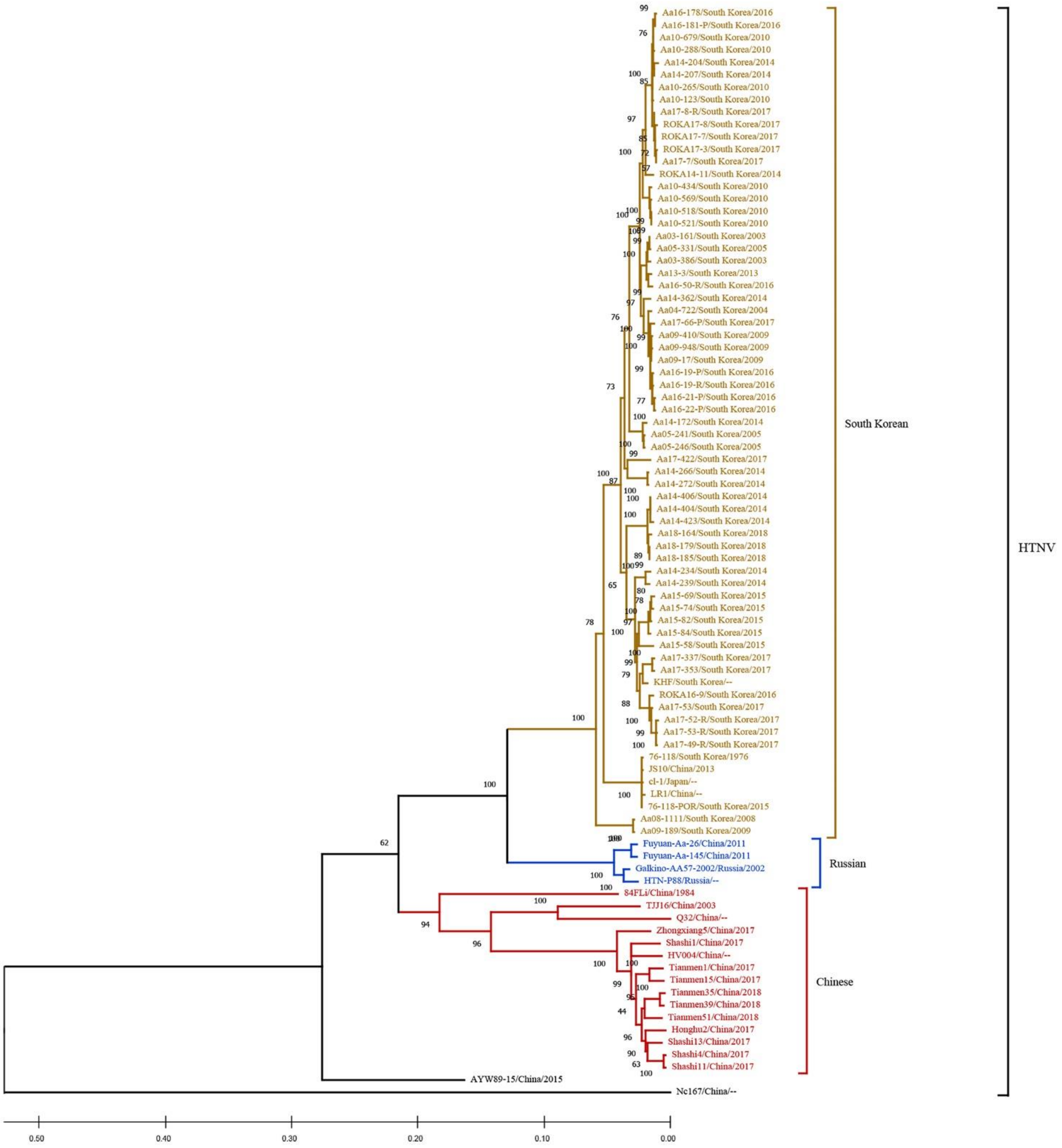

SF1. Maximum Likelihood Tree (ML) of Hantaan virus (HTNV) strains. South Korean, first clade; Russian, second clade; Chinese, third clade; (--), Not Available. Each taxon is displayed as follows: Strain name/Country/Collection year. The concatenated coding sequences of each strain were used to construct the ML tree.

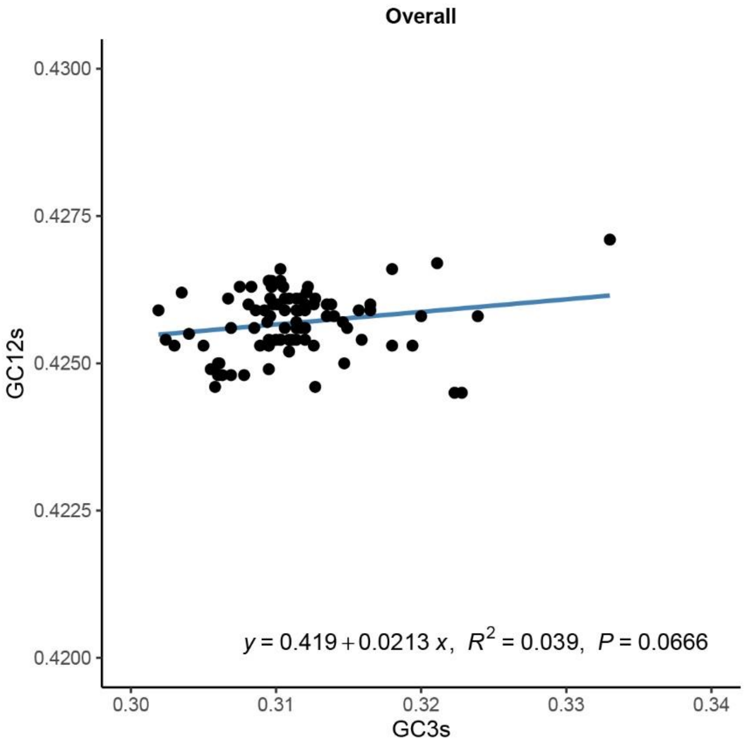

SF2. Neutrality plot analysis in HTNV overall genome for all strains.
