# Supplementary material for: Edging on Mutational Bias, Induced Natural Selection From Host and Natural Reservoirs Predominates Codon Usage Evolution in Hantaan Virus
Source: Front Microbiol. 2021 Jul 2;12:699788. doi: 10.3389/fmicb.2021.699788 (PMC8283416; doi:10.3389/fmicb.2021.699788)
Supplement: Supplementary file 2 [file Data_Sheet_2.PDF]

ST1. Hantaan virus strains information.

S. No	Isolation	GenBank Accession Number			Host & Reservoir	Phylogenetic Clade	Recombinants S.
		L	M	S			
1	Nc167	DQ989237.1	AB027115.2	AB027523.1	Niviventer confucianus	X	
2	AYW89-15	KY978757.1	KY978756.1	KY978755.1	Apodemus agrarius	X	
3	Fuyuan-Aa-26	KJ857317.1	KJ857334.1	KJ857347.1	Apodemus agrarius	Russian	
4	Fuyuan-Aa-145	KJ857319.1	KJ857336.1	KJ857349.1	Apodemus agrarius	Russian	
5	Galkino/AA57/2002	AB620033.1	AB620032.1	AB620031.1	Apodemus agrarius	Russian	
6	HTN-P88	MH251330.1	MH251329.1	MH251328.1	Homo sapiens	Russian	
7	Aa08-1111	KY594712.1	KY594715.1	KY594718.1	Apodemus agrarius	South Korean	
8	Aa09-189	KY594713.1	KY594716.1	KY594719.1	Apodemus agrarius	South Korean	
9	LR1	AF288292.1	AF288293.1	AF288294.1	--	South Korean	
10	76-118	X55901.1	Y00386.1	M14626.1	Apodemus agrarius	South Korean	
11	JS10	KP896316.1	KP970569.1	KP970581.1	Homo sapiens	South Korean	
12	76-118/POR	KT885047.1	KT885048.1	KT885049.1	Apodemus agrarius	South Korean	
13	cl-1	D25528.1	D25529.1	D25530.1	--	South Korean	
14	Aa14-266	KT934979.1	KT935013.1	KT935047.1	--	South Korean	
15	Aa14-272	KT934980.1	KT935014.1	KT935048.1	--	South Korean	
16	Aa17-422	MT012578.1	MT012566.1	MT012554.1	Apodemus agrarius	South Korean	
17	Aa05-241	KT934960.1	KT934994.1	KT935028.1	--	South Korean	
18	Aa05-246	KU207175.1	KU207183.1	KU207191.1	Apodemus agrarius	South Korean	
19	Aa14-172	KT934974.1	KT935008.1	KT935042.1	--	South Korean	
20	Aa10-434	KT934970.1	KT935004.1	KT935038.1	--	South Korean	
21	Aa10-569	MH598472.1	MH598486.1	MH598500.1	Apodemus agrarius	South Korean	
22	Aa10-518	KT934971.1	KT935005.1	KT935039.1	--	South Korean	
23	Aa10-521	MH598471.1	MH598485.1	MH598499.1	Apodemus agrarius	South Korean	
24	ROKA14-11	KU207199.1	KU207203.1	KU207207.1	Homo sapiens	South Korean	
25	Aa14-204	KU207159.1	KU207166.1	KU207173.1	Apodemus agrarius	South Korean	
26	ROKA17-3	MH598467.1	MH598481.1	MH598495.1	Homo sapiens	South Korean	
27	Aa17-8-R	MK548695.1	MK548686.1	MK548677.1	Apodemus agrarius	South Korean	
28	ROKA17-8	MH598470.1	MH598484.1	MH598498.1	Homo sapiens	South Korean	
29	Aa17-7	MH598475.1	MH598489.1	MH598503.1	Apodemus agrarius	South Korean	
30	ROKA17-7	MH598469.1	MH598483.1	MH598497.1	Homo sapiens	South Korean	
31	Aa16-178	MH598474.1	MH598488.1	MH598502.1	Apodemus agrarius	South Korean	
32	Aa16-181-P	MK548668.1	MK548659.1	MK548650.1	Apodemus agrarius	South Korean	
33	Aa14-207	KT934978.1	KT935012.1	KT935046.1	--	South Korean	
34	Aa10-123	KT934968.1	KT935002.1	KT935036.1	--	South Korean	
35	Aa10-265	KU207178.1	KU207186.1	KU207194.1	Apodemus agrarius	South Korean	
36	Aa10-288	KT934969.1	KT935003.1	KT935037.1	--	South Korean	
37	Aa10-679	KT934973.1	KT935007.1	KT935041.1	--	South Korean	
38	Aa03-386	KT934957.1	KT934991.1	KT935025.1	--	South Korean	
39	Aa03-161	KT934956.1	KT934990.1	KT935024.1	--	South Korean	
40	Aa05-331	KT934962.1	KT934996.1	KT935030.1	--	South Korean	
41	Aa13-3	KX687237.1	KX687227.1	KX687232.1	--	South Korean	
42	Aa16-50-R	MK548692.1	MK548683.1	MK548674.1	Apodemus agrarius	South Korean	
43	Aa14-362	KT934981.1	KT935015.1	KT935049.1	--	South Korean	
44	Aa04-722	KU207174.1	KU207182.1	KU207190.1	Apodemus agrarius	South Korean	
45	Aa17-66-P	MK548671.1	MK548662.1	MK548653.1	Apodemus agrarius	South Korean	
46	Aa16-21-P	MK548665.1	MK548656.1	MK548647.1	Apodemus agrarius	South Korean	
47	Aa16-22-P	MK548666.1	MK548657.1	MK548648.1	Apodemus agrarius	South Korean	

48	Aa09-410	KU207177.1	KU207185.1	KU207193.1	Apodemus agrarius	South Korean	
49	Aa09-948	KT934966.1	KT935000.1	KT935034.1	--	South Korean	
50	Aa09-17	KU207176.1	KU207184.1	KU207192.1	Apodemus agrarius	South Korean	
51	Aa16-19-P	MK548664.1	MK548655.1	MK548646.1	Apodemus agrarius	South Korean	
52	Aa16-19-R	MK548691.1	MK548682.1	MK548673.1	Apodemus agrarius	South Korean	
53	Aa14-423	KT934988.1	KT935022.1	KT935056.1	--	South Korean	
54	Aa14-406	KT934985.1	KT935019.1	KT935053.1	--	South Korean	
55	Aa14-404	KT934984.1	KT935018.1	KT935052.1	--	South Korean	
56	Aa18-164	MT012579.1	MT012567.1	MT012555.1	Apodemus agrarius	South Korean	
57	Aa18-179	MT012580.1	MT012568.1	MT012556.1	Apodemus agrarius	South Korean	
58	Aa18-185	MT012581.1	MT012569.1	MT012557.1	Apodemus agrarius	South Korean	
59	Aa15-58	KU207180.1	KU207188.1	KU207196.1	Apodemus agrarius	South Korean	
60	Aa14-234	KX687240.1	KX687230.1	KX687235.1	--	South Korean	
61	Aa14-239	KX687241.1	KX687231.1	KX687236.1	--	South Korean	
62	Aa15-84	MT012573.1	MT012561.1	MT012549.1	Apodemus agrarius	South Korean	
63	Aa15-69	MT012570.1	MT012558.1	MT012546.1	Apodemus agrarius	South Korean	
64	Aa15-74	MT012571.1	MT012559.1	MT012547.1	Apodemus agrarius	South Korean	
65	Aa15-82	MT012572.1	MT012560.1	MT012548.1	Apodemus agrarius	South Korean	
66	ROKA16-9	MH598466.1	MH598480.1	MH598494.1	Homo sapiens	South Korean	
67	Aa17-53	MH598479.1	MH598493.1	MH598507.1	Apodemus agrarius	South Korean	
68	Aa17-52-R	MK548698.1	MK548689.1	MK548680.1	Apodemus agrarius	South Korean	
69	Aa17-53-R	MK548699.1	MK548690.1	MK548681.1	Apodemus agrarius	South Korean	
70	Aa17-49-R	MK548697.1	MK548688.1	MK548679.1	Apodemus agrarius	South Korean	
71	KHF	LC209210.1	LC209208.1	LC209206.1	Homo sapiens	South Korean	
72	Aa17-337	MT012574.1	MT012562.1	MT012550.1	Apodemus agrarius	South Korean	
73	Aa17-353	MT012575.1	MT012563.1	MT012551.1	Apodemus agrarius	South Korean	
74	84FLi	AF336826.1	AF345636.2	AY017064.1	Homo sapiens	Chinese	
75	Zhongxiang5	MN608085.1	MN608074.1	MN608063.1	Apodemus agrarius	Chinese	
76	Shashi1	MN608080.1	MN608069.1	MN608058.1	Apodemus agrarius	Chinese	
77	HV004	JQ083393.1	JQ083394.1	JQ083395.1	Apodemus agrarius	Chinese	
78	Tianmen1	MN608086.1	MN608075.1	MN608064.1	Homo sapiens	Chinese	
79	Tianmen15	MN608087.1	MN608076.1	MN608065.1	Homo sapiens	Chinese	
80	Honghu2	MN608084.1	MN608073.1	MN608062.1	Apodemus agrarius	Chinese	
81	Shashi13	MN608083.1	MN608072.1	MN608060.1	Apodemus agrarius	Chinese	
82	Shashi4	MN608081.1	MN608070.1	MN608059.1	Apodemus agrarius	Chinese	
83	Shashi11	MN608082.1	MN608071.1	MN608061.1	Apodemus agrarius	Chinese	
84	Tianmen51	MN608090.1	MN608079.1	MN608068.1	Homo sapiens	Chinese	
85	Tianmen35	MN608088.1	MN608077.1	MN608066.1	Homo sapiens	Chinese	
86	Tianmen39	MN608089.1	MN608078.1	MN608067.1	Homo sapiens	Chinese	
87	TJJ16	KU215675.1	EU074672.1	AY839871.1	Niviventer confucianus	Chinese	
88	Q32	DQ371906.1	DQ371905.1	AB027097.1	Apodemus agrarius	Chinese	
89	XAAa10091712	JN542542.1			Apodemus agrarius		Recombinants
90	ROKA13-8	KU207198.1	KU207202.1	KU207206.1	Homo sapiens		Recombinants
91	Aa14-175	KU207156.1	KU207163.1	KU207170.1	Apodemus agrarius		Recombinants
92	Aa14-184	KU207157.1	KU207164.1	KU207171.1	Apodemus agrarius		Recombinants
93	Aa14-188	KU207158.1	KU207165.1	KU207172.1	Apodemus agrarius		Recombinants
94	Aa14-186	KX687238.1	KX687228.1	KX687233.1	Apodemus agrarius		Recombinants
95	ROKA17-5	MH598468.1	MH598482.1	MH598496.1	Homo sapiens		Recombinants

L, L-segment; M, M-segment; S, S-segment; (--), Not Available; X, isolate regarded as out of the three phylogenetic clades.

ST2. Nucleotide composition analysis of HTNV coding sequences (%).

SEQ/PAR	A	U	G	C	GC	AU	GC1	GC2	GC12	A3	U3	G3	C3	GC3	AU3	Gravy	ARO
Nc167	31.13	29.07	22.39	17.42	39.80	60.20	48.37	37.06	42.71	31.11	35.60	17.88	17.88	33.30	66.70	-0.16	0.10
AYW89-15	32.24	28.33	21.87	17.56	39.43	60.57	47.77	37.38	42.58	33.57	34.04	16.39	16.39	32.39	67.61	-0.17	0.10
Fuyuan-Aa-26	32.33	28.93	21.73	17.01	38.74	61.26	47.51	37.55	42.53	34.23	35.47	15.89	15.89	30.30	69.70	-0.16	0.10
Fuyuan-Aa-145	32.30	28.96	21.73	17.01	38.73	61.27	47.63	37.46	42.54	34.23	35.52	15.84	15.84	30.24	69.76	-0.16	0.10
Galkino-AA57-2002	32.25	28.87	21.83	17.05	38.89	61.11	47.67	37.45	42.56	34.00	35.32	16.28	16.28	30.69	69.31	-0.16	0.10
HTN-P88	32.31	28.89	21.88	16.92	38.80	61.20	47.80	37.45	42.62	33.91	35.74	16.27	16.27	30.35	69.65	-0.17	0.10
Aa08-1111	32.32	28.55	21.91	17.22	39.13	60.87	47.61	37.56	42.58	34.13	34.47	16.35	16.35	31.40	68.60	-0.16	0.10
Aa09-189	32.30	28.57	21.91	17.21	39.13	60.87	47.64	37.56	42.60	34.10	34.52	16.35	16.35	31.38	68.62	-0.16	0.10
LR1	32.44	28.65	21.79	17.12	38.91	61.09	47.51	37.71	42.61	34.85	34.48	15.84	15.84	30.67	69.33	-0.16	0.10
76-118	32.52	28.66	21.74	17.08	38.82	61.18	47.39	37.62	42.50	34.87	34.53	15.86	15.86	30.60	69.40	-0.16	0.10
JS10	32.53	28.67	21.74	17.07	38.80	61.20	47.36	37.59	42.48	34.87	34.53	15.81	15.81	30.60	69.40	-0.15	0.10
76-118-POR	32.54	28.67	21.72	17.07	38.79	61.21	47.37	37.55	42.46	34.91	34.51	15.81	15.81	30.58	69.42	-0.15	0.10
cl-1	32.54	28.66	21.72	17.07	38.79	61.21	47.40	37.57	42.49	34.93	34.51	15.78	15.78	30.55	69.45	-0.15	0.10
Aa14-266	32.62	28.54	21.72	17.12	38.83	61.17	47.41	37.55	42.48	35.05	34.26	15.74	15.74	30.69	69.31	-0.16	0.10
Aa14-272	32.63	28.51	21.70	17.16	38.86	61.14	47.41	37.55	42.48	35.10	34.12	15.71	15.71	30.78	69.22	-0.16	0.10
Aa17-422	32.59	28.49	21.77	17.15	38.92	61.08	47.37	37.60	42.49	34.93	34.12	16.01	16.01	30.95	69.05	-0.16	0.10
Aa05-241	32.53	28.66	21.73	17.07	38.81	61.19	47.40	37.66	42.53	34.91	34.60	15.87	15.87	30.50	69.50	-0.16	0.10
Aa05-246	32.54	28.63	21.73	17.10	38.82	61.18	47.37	37.63	42.50	34.91	34.48	15.87	15.87	30.61	69.39	-0.16	0.10
Aa14-172	32.48	28.70	21.78	17.03	38.81	61.19	47.36	37.59	42.48	34.73	34.64	16.03	16.03	30.63	69.37	-0.16	0.10
Aa10-434	32.29	28.57	21.92	17.21	39.14	60.86	47.42	37.72	42.57	34.33	34.21	16.29	16.29	31.46	68.54	-0.15	0.10
Aa10-569	32.34	28.50	21.91	17.25	39.16	60.84	47.43	37.66	42.54	34.34	34.06	16.34	16.34	31.59	68.41	-0.16	0.10
Aa10-518	32.31	28.48	21.94	17.27	39.21	60.79	47.46	37.71	42.59	34.29	34.06	16.37	16.37	31.65	68.35	-0.16	0.10
Aa10-521	32.32	28.46	21.93	17.29	39.22	60.78	47.49	37.71	42.60	34.32	34.04	16.34	16.34	31.65	68.35	-0.16	0.10
ROKA14-11	32.40	28.50	21.84	17.26	39.10	60.90	47.51	37.71	42.61	34.56	34.17	16.04	16.04	31.27	68.73	-0.16	0.10
Aa14-204	32.45	28.51	21.83	17.21	39.05	60.95	47.49	37.57	42.53	34.57	34.18	16.15	16.15	31.26	68.74	-0.16	0.10
ROKA17-3	32.38	28.61	21.85	17.16	39.01	60.99	47.49	37.60	42.54	34.48	34.40	16.18	16.18	31.11	68.89	-0.16	0.10
Aa17-8-R	32.41	28.61	21.84	17.14	38.98	61.02	47.49	37.60	42.54	34.57	34.40	16.12	16.12	31.03	68.97	-0.16	0.10
ROKA17-8	32.36	28.60	21.89	17.15	39.04	60.96	47.49	37.60	42.54	34.43	34.37	16.26	16.26	31.20	68.80	-0.16	0.10
Aa17-7	32.37	28.62	21.86	17.14	39.00	61.00	47.49	37.60	42.54	34.46	34.46	16.18	16.18	31.09	68.91	-0.16	0.10
ROKA17-7	32.36	28.61	21.88	17.15	39.03	60.97	47.51	37.60	42.56	34.46	34.40	16.20	16.20	31.14	68.86	-0.16	0.10
Aa16-178	32.35	28.59	21.89	17.18	39.07	60.93	47.54	37.63	42.59	34.43	34.37	16.23	16.23	31.20	68.80	-0.16	0.10
Aa16-181-P	32.37	28.61	21.85	17.17	39.02	60.98	47.54	37.63	42.59	34.51	34.43	16.12	16.12	31.06	68.94	-0.16	0.10
Aa14-207	32.43	28.53	21.84	17.20	39.05	60.95	47.51	37.60	42.56	34.63	34.18	16.12	16.12	31.20	68.80	-0.16	0.10
Aa10-123	32.40	28.61	21.85	17.13	38.98	61.02	47.43	37.60	42.52	34.51	34.40	16.18	16.18	31.09	68.91	-0.16	0.10
Aa10-265	32.39	28.57	21.86	17.18	39.04	60.96	47.54	37.60	42.57	34.51	34.34	16.18	16.18	31.14	68.86	-0.16	0.10
Aa10-288	32.40	28.58	21.85	17.17	39.02	60.98	47.51	37.57	42.54	34.51	34.34	16.15	16.15	31.14	68.86	-0.16	0.10
Aa10-679	32.42	28.58	21.83	17.17	39.00	61.00	47.51	37.60	42.56	34.60	34.34	16.09	16.09	31.06	68.94	-0.16	0.10
Aa03-386	32.46	28.56	21.78	17.20	38.98	61.02	47.57	37.69	42.63	34.85	34.32	15.84	15.84	30.83	69.17	-0.16	0.10
Aa03-161	32.42	28.56	21.82	17.20	39.02	60.98	47.57	37.71	42.64	34.77	34.29	15.92	15.92	30.95	69.05	-0.16	0.10
Aa05-331	32.40	28.55	21.83	17.21	39.05	60.95	47.57	37.71	42.64	34.71	34.26	15.95	15.95	31.03	68.97	-0.16	0.10
Aa13-3	32.39	28.55	21.86	17.20	39.06	60.94	47.56	37.62	42.59	34.64	34.19	16.09	16.09	31.16	68.84	-0.16	0.10
Aa16-50-R	32.43	28.54	21.81	17.22	39.03	60.97	47.57	37.71	42.64	34.82	34.20	15.84	15.84	30.97	69.03	-0.16	0.10
Aa14-362	32.52	28.51	21.79	17.19	38.98	61.02	47.47	37.68	42.57	34.98	34.08	15.92	15.92	30.94	69.06	-0.16	0.10
Aa04-722	32.49	28.57	21.80	17.14	38.94	61.06	47.42	37.70	42.56	34.87	34.28	16.00	16.00	30.85	69.15	-0.16	0.10
Aa17-66-P	32.42	28.61	21.85	17.12	38.98	61.02	47.46	37.71	42.59	34.68	34.40	16.09	16.09	30.92	69.08	-0.16	0.10
Aa16-21-P	32.47	28.54	21.83	17.15	38.98	61.02	47.40	37.69	42.54	34.79	34.18	16.06	16.06	31.03	68.97	-0.16	0.10
Aa16-22-P	32.48	28.59	21.79	17.14	38.93	61.07	47.37	37.69	42.53	34.82	34.29	15.92	15.92	30.89	69.11	-0.16	0.10
Aa09-410	32.47	28.57	21.78	17.18	38.96	61.04	47.49	37.69	42.59	34.82	34.32	15.89	15.89	30.86	69.14	-0.16	0.10
Aa09-948	32.46	28.59	21.80	17.15	38.95	61.05	47.37	37.69	42.53	34.74	34.32	16.01	16.01	30.95	69.05	-0.16	0.10
Aa09-17	32.47	28.58	21.79	17.16	38.95	61.05	47.40	37.66	42.53	34.77	34.29	15.98	15.98	30.95	69.05	-0.16	0.10
Aa16-19-P	32.40	28.55	21.87	17.18	39.05	60.95	47.43	37.69	42.56	34.57	34.23	16.20	16.20	31.20	68.80	-0.16	0.10
Aa16-19-R	32.45	28.58	21.82	17.15	38.98	61.02	47.40	37.69	42.54	34.74	34.26	16.06	16.06	31.00	69.00	-0.16	0.10
Aa14-423	32.69	28.36	21.63	17.32	38.95	61.05	47.61	37.58	42.60	35.45	33.74	15.42	15.42	30.81	69.19	-0.16	0.10

Aa14-406	32.58	28.42	21.70	17.30	39.00	61.00	47.56	37.64	42.60	35.14	33.88	15.62	15.62	30.98	69.02	-0.16	0.10
Aa14-404	32.60	28.39	21.70	17.31	39.01	60.99	47.56	37.64	42.60	35.17	33.82	15.62	15.62	31.01	68.99	-0.16	0.10
Aa18-164	32.56	28.39	21.70	17.35	39.05	60.95	47.54	37.63	42.59	35.05	33.81	15.67	15.67	31.14	68.86	-0.16	0.10
Aa18-179	32.53	28.39	21.73	17.35	39.07	60.93	47.54	37.66	42.60	34.99	33.81	15.73	15.73	31.20	68.80	-0.16	0.10
Aa18-185	32.53	28.37	21.73	17.37	39.09	60.91	47.54	37.66	42.60	34.99	33.75	15.73	15.73	31.26	68.74	-0.16	0.10
Aa15-58	32.67	28.37	21.65	17.31	38.96	61.04	47.49	37.60	42.54	35.24	33.81	15.59	15.59	30.95	69.05	-0.16	0.10
Aa14-234	32.53	28.45	21.76	17.25	39.01	60.99	47.51	37.71	42.61	35.01	34.03	15.73	15.73	30.96	69.04	-0.16	0.10
Aa14-239	32.52	28.44	21.78	17.26	39.04	60.96	47.57	37.66	42.61	34.96	33.98	15.78	15.78	31.06	68.94	-0.15	0.10
Aa15-84	32.45	28.56	21.79	17.20	38.99	61.01	47.56	37.61	42.58	34.75	34.30	15.90	15.90	30.96	69.04	-0.16	0.10
Aa15-69	32.45	28.53	21.83	17.19	39.02	60.98	47.54	37.71	42.63	34.79	34.23	15.98	15.98	30.97	69.03	-0.16	0.10
Aa15-74	32.39	28.55	21.87	17.19	39.06	60.94	47.60	37.71	42.66	34.65	34.32	16.09	16.09	31.03	68.97	-0.16	0.10
Aa15-82	32.45	28.48	21.84	17.23	39.07	60.93	47.57	37.66	42.61	34.74	34.09	16.06	16.06	31.17	68.83	-0.16	0.10
ROKA16-9	32.53	28.42	21.78	17.27	39.05	60.95	47.54	37.69	42.61	34.91	34.01	15.98	15.98	31.09	68.91	-0.16	0.10
Aa17-53	32.52	28.42	21.80	17.27	39.07	60.93	47.49	37.74	42.61	34.93	33.92	15.95	15.95	31.14	68.86	-0.16	0.10
Aa17-52-R	32.57	28.48	21.76	17.19	38.95	61.05	47.51	37.74	42.63	35.13	34.12	15.84	15.84	30.75	69.25	-0.16	0.10
Aa17-53-R	32.53	28.45	21.80	17.22	39.02	60.98	47.54	37.74	42.64	35.02	34.04	15.89	15.89	30.95	69.05	-0.16	0.10
Aa17-49-R	32.54	28.41	21.79	17.26	39.05	60.95	47.50	37.76	42.63	35.01	33.94	15.95	15.95	31.05	68.95	-0.16	0.10
KHF	32.72	28.49	21.63	17.16	38.79	61.21	47.42	37.68	42.55	35.49	34.11	15.47	15.47	30.40	69.60	-0.16	0.10
Aa17-337	32.56	28.34	21.74	17.36	39.10	60.90	47.56	37.70	42.63	35.09	33.69	15.81	15.81	31.22	68.78	-0.15	0.10
Aa17-353	32.62	28.34	21.68	17.36	39.04	60.96	47.49	37.63	42.56	35.16	33.67	15.70	15.70	31.17	68.83	-0.16	0.10
84FLi	32.55	28.70	21.56	17.19	38.74	61.26	47.82	37.35	42.59	34.79	35.02	15.42	15.42	30.19	69.81	-0.17	0.10
Zhongxiang5	31.93	28.76	21.96	17.36	39.32	60.68	47.57	37.34	42.45	32.79	34.98	16.69	16.69	32.23	67.77	-0.16	0.10
Shashi1	31.93	28.84	21.97	17.26	39.23	60.77	47.60	37.47	42.53	32.98	35.22	16.62	16.62	31.80	68.20	-0.16	0.10
HV004	31.87	28.80	21.89	17.44	39.33	60.67	47.57	37.33	42.45	32.73	35.00	16.53	16.53	32.28	67.72	-0.16	0.10
Tianmen1	32.13	28.56	21.76	17.55	39.31	60.69	47.88	37.44	42.66	33.71	34.49	15.89	15.89	31.80	68.20	-0.16	0.10
Tianmen15	32.01	28.58	21.93	17.48	39.42	60.58	47.91	37.44	42.67	33.37	34.52	16.36	16.36	32.11	67.89	-0.16	0.10
Honghu2	32.08	28.73	21.81	17.38	39.19	60.81	47.77	37.41	42.59	33.57	34.86	16.03	16.03	31.57	68.43	-0.16	0.10
Shashi13	32.03	28.70	21.85	17.42	39.27	60.73	47.60	37.47	42.53	33.26	34.80	16.31	16.31	31.94	68.06	-0.17	0.10
Shashi4	32.10	28.75	21.82	17.32	39.15	60.85	47.68	37.44	42.56	33.51	35.00	16.25	16.25	31.49	68.51	-0.17	0.10
Shashi11	32.11	28.78	21.82	17.29	39.11	60.89	47.74	37.41	42.58	33.60	35.05	16.17	16.17	31.35	68.65	-0.16	0.10
Tianmen51	32.07	28.61	21.82	17.49	39.32	60.68	47.68	37.47	42.58	33.48	34.52	16.17	16.17	32.00	68.00	-0.17	0.10
Tianmen35	32.01	28.90	21.80	17.29	39.09	60.91	47.77	37.47	42.62	33.37	35.42	16.03	16.03	31.21	68.79	-0.16	0.10
Tianmen39	32.03	28.84	21.77	17.36	39.13	60.87	47.77	37.44	42.60	33.43	35.22	16.00	16.00	31.35	68.65	-0.16	0.10
TJJ16	32.31	28.68	21.67	17.34	39.01	60.99	47.50	37.42	42.46	33.80	34.92	15.92	15.92	31.27	68.73	-0.17	0.10
Q32	32.24	28.62	21.73	17.40	39.14	60.86	47.64	37.51	42.50	33.91	34.62	16.03	15.44	31.47	68.53	-0.17	0.10
**Mean ±**	32.38	28.59	21.81	17.22	39.03	60.97	47.55	37.59	42.57	34.47	34.40	16.03	16.02	31.13	68.87	-0.16	0.10
**STD**	0.23	0.15	0.10	0.12	0.17	0.17	0.15	0.12	0.05	0.69	0.45	0.32	0.32	0.49	0.49	0.00	0.00

Gravy, protein's general average hydropathicity; ARO, protein's aromaticity; STD, standard deviation.

ST3. RSCU patterns of HTNV strains (based on isolation host) with its relevant host and natural reservoirs.

AA	Codons	HTNV-HS	HS	HTNV-APO	APO	HTNV-NC	NC
Phe(F)	UUU	**1.32**	0.99	**1.33**	0.90	**1.40**	0.86
	UUC	0.68	**1.01**	0.67	**1.10**	0.60	**1.14**
Leu(L)	UUA	**1.84**	0.54	**1.84**	0.26	**1.77**	0.14
	UUG	0.94	0.83	0.97	0.69	0.99	0.59
	CUU	1.02	0.87	1.01	0.58	1.06	0.41
	CUC	0.48	1.09	0.46	1.53	0.51	1.60
	CUA	0.97	0.46	0.96	0.57	0.91	0.53
	CUG	0.74	**2.21**	0.76	**2.39**	0.75	**2.73**
Ile(I)	AUU	**1.36**	1.15	**1.37**	0.69	**1.41**	0.76
	AUC	0.58	**1.29**	0.59	**1.87**	0.65	**1.87**
	AUA	1.06	0.56	1.05	0.43	0.94	0.37
Val(V)	GUU	**1.41**	0.80	**1.37**	0.35	**1.64**	0.26

	GUC	0.66	0.92	0.65	0.99	0.52	0.81
	GUA	1.13	0.53	1.22	0.45	1.00	0.40
	GUG	0.81	**1.75**	0.76	**2.21**	0.85	**2.52**
Ser(S)	UCU	1.29	1.18	1.29	1.39	1.34	1.06
	UCC	0.51	1.20	0.52	1.45	0.55	**1.80**
	UCA	**2.05**	0.99	**2.03**	0.62	**2.16**	0.65
	UCG	0.13	0.28	0.12	0.40	0.11	0.34
	AGU	1.16	0.98	1.17	0.65	1.13	0.97
	AGC	0.86	**1.37**	0.87	**1.49**	0.71	1.18
Pro(P)	CCU	**1.70**	**1.22**	1.67	1.29	1.58	**1.54**
	CCC	0.39	1.20	0.39	**1.58**	0.35	1.42
	CCA	**1.70**	1.19	**1.75**	0.82	**1.77**	0.92
	CCG	0.21	0.39	0.20	0.30	0.31	0.12
Thr(T)	ACU	0.94	1.06	0.85	0.83	0.92	0.56
	ACC	0.35	**1.31**	0.36	**1.50**	0.47	**1.93**
	ACA	**2.63**	1.22	**2.72**	1.07	**2.52**	0.97
	ACG	0.08	0.41	0.08	0.60	0.10	0.54
Ala(A)	GCU	1.18	1.12	1.20	1.15	1.19	1.17
	GCC	0.57	**1.52**	0.57	**1.85**	0.59	**1.98**
	GCA	**2.20**	1.01	**2.17**	0.84	**2.14**	0.73
	GCG	0.05	0.35	0.07	0.16	0.07	0.12
Tyr(Y)	UAU	**1.42**	0.95	**1.40**	0.72	**1.45**	0.85
	UAC	0.58	**1.05**	0.61	**1.28**	0.55	**1.15**
His(H)	CAU	**1.36**	0.90	**1.31**	0.56	**1.39**	0.70
	CAC	0.64	**1.10**	0.69	**1.44**	0.61	**1.30**
Gln(Q)	CAA	**1.03**	0.57	**1.08**	0.42	0.99	0.38
	CAG	0.97	**1.43**	0.92	**1.58**	**1.01**	**1.62**
Asn(N)	AAU	**1.43**	**1.01**	**1.45**	0.71	**1.41**	0.75
	AAC	0.57	0.99	0.55	**1.29**	0.60	**1.25**
Lys(K)	AAA	**1.03**	0.93	**1.03**	0.67	**1.05**	0.55
	AAG	0.97	**1.07**	0.98	**1.33**	0.96	**1.45**
Asp(D)	GAU	**1.43**	1.00	**1.44**	0.73	**1.38**	0.58
	GAC	0.57	1.00	0.56	**1.27**	0.62	**1.42**
Glu(E)	GAA	**1.18**	0.92	**1.13**	0.74	**1.04**	0.84
	GAG	0.82	**1.08**	0.87	**1.26**	0.97	**1.16**
Cys(C)	UGU	**1.40**	0.98	**1.39**	**1.42**	**1.29**	**1.21**
	UGC	0.60	**1.02**	0.61	0.58	0.71	0.79
Arg(R)	CGU	0.47	0.49	0.50	0.58	0.53	0.48
	CGC	0.28	0.94	0.22	0.80	0.11	0.52
	CGA	0.47	0.69	0.52	0.71	0.50	0.94
	CGG	0.43	1.14	0.41	1.22	0.55	0.92
	AGA	**2.34**	**1.44**	**2.34**	1.27	**2.22**	1.50
	AGG	2.01	1.31	2.01	**1.42**	2.09	**1.65**
Gly(G)	GGU	**1.24**	0.69	1.23	0.54	**1.23**	0.79
	GGC	0.42	**1.25**	0.43	**1.54**	0.59	**1.46**
	GGA	**1.24**	1.09	**1.25**	0.87	1.07	0.75
	GGG	1.10	0.97	1.10	1.06	1.11	1.00

RSCU, Relative synonymous codon usage; HTNV-HS, HTNV strains isolated from H. sapiens (14 strains); HTNV-APO, HTNV strains isolated from A. agrarius (49 strains); HTNV-NC, HTNV strains isolated from N. confucianus (two strains); HS, H. sapiens; APO, A. agrarius; NC, N. confucianus. Bold represents preferred codons by HTNV, host, and natural reservoirs.

ST4. Effective number of codon analysis (ENC) of HTNV strains.

**All Strains**	**ENC**			
	**Overall**	**L-segment**	**M-segment**	**S-segment**
Nc167	48.74	47.53	49.39	52.01
AYW89-15	47.71	46.83	48.68	48.76
Fuyuan-Aa-26	47.34	45.22	49.31	49.38
Fuyuan-Aa-145	47.55	45.53	49.34	49.61

Galkino-AA57-2002	47.31	45.07	49.46	48.84
HTN-P88	47.23	44.82	49.93	48.33
Aa08-1111	47.87	46.66	48.34	50.36
Aa09-189	47.94	46.81	48.32	50.42
LR1	47.49	45.91	48.88	49.68
76-118	47.29	45.85	48.32	49.50
JS10	47.32	45.80	48.53	49.47
76-118-POR	47.27	45.78	48.33	49.76
cl-1	47.31	45.84	48.34	49.76
Aa14-266	46.97	45.56	48.30	48.15
Aa14-272	47.08	45.74	48.51	47.85
Aa17-422	47.36	45.84	48.42	48.58
Aa05-241	47.31	45.26	49.71	50.50
Aa05-246	47.37	45.24	49.75	50.64
Aa14-172	47.32	45.21	49.80	50.85
Aa10-434	47.43	45.52	49.74	49.84
Aa10-569	47.53	45.68	49.86	49.69
Aa10-518	47.54	45.68	49.84	49.69
Aa10-521	47.53	45.64	49.86	49.69
ROKA14-11	47.61	45.91	49.92	49.46
Aa14-204	47.42	45.48	49.94	50.19
ROKA17-3	47.40	45.49	49.73	49.96
Aa17-8-R	47.36	45.42	49.68	50.30
ROKA17-8	47.52	45.60	49.97	50.18
Aa17-7	47.43	45.56	49.72	50.38
ROKA17-7	47.44	45.60	49.73	50.11
Aa16-178	47.48	45.54	49.98	49.94
Aa16-181-P	47.41	45.40	49.90	50.16
Aa14-207	47.42	45.36	49.99	50.19
Aa10-123	47.33	45.26	49.92	50.19
Aa10-265	47.40	45.34	49.86	50.47
Aa10-288	47.38	45.34	49.91	50.19
Aa10-679	47.33	45.25	49.87	50.19
Aa03-386	47.41	45.68	49.52	49.40
Aa03-161	47.45	45.68	49.53	49.67
Aa05-331	47.49	45.74	49.56	49.67
Aa13-3	47.55	45.91	49.41	49.81
Aa16-50-R	47.31	45.49	49.49	50.11
Aa14-362	47.34	45.58	49.55	48.67
Aa04-722	47.29	45.47	49.61	48.64
Aa17-66-P	47.28	45.55	49.73	48.54
Aa16-21-P	47.35	45.70	49.52	48.93
Aa16-22-P	47.22	45.42	49.51	49.00
Aa09-410	47.32	45.61	49.61	48.87
Aa09-948	47.34	45.61	49.65	48.87
Aa09-17	47.36	45.63	49.61	48.93
Aa16-19-P	47.44	45.76	49.75	49.05
Aa16-19-R	47.30	45.61	49.59	48.95
Aa14-423	47.61	45.16	50.07	50.23
Aa14-406	47.67	45.42	49.82	49.96
Aa14-404	47.69	45.45	49.82	49.96
Aa18-164	47.71	45.64	49.71	50.22

Aa18-179	47.79	45.77	49.84	49.92
Aa18-185	47.77	45.68	49.89	49.96
Aa15-58	47.58	46.14	49.41	48.94
Aa14-234	47.33	45.52	49.48	48.61
Aa14-239	47.63	46.12	49.35	48.89
Aa15-84	47.61	46.02	49.38	49.24
Aa15-69	47.62	45.63	50.13	49.18
Aa15-74	47.72	45.81	50.15	49.18
Aa15-82	47.81	45.93	50.19	49.24
ROKA16-9	47.56	46.11	49.46	49.90
Aa17-53	47.63	46.15	49.60	49.81
Aa17-52-R	47.41	46.05	48.98	49.81
Aa17-53-R	47.51	46.06	49.32	49.81
Aa17-49-R	47.60	46.11	49.55	49.81
KHF	47.30	45.56	49.45	49.93
Aa17-337	47.59	45.82	49.77	49.90
Aa17-353	47.66	45.86	49.94	49.97
84FLi	47.36	46.58	46.87	51.40
Zhongxiang5	47.86	47.65	47.52	51.10
Shashi1	47.73	47.32	47.40	50.62
HV004	48.03	47.67	47.33	52.50
Tianmen1	47.94	47.45	47.45	50.42
Tianmen15	47.91	47.67	47.57	49.70
Honghu2	47.74	47.06	47.03	50.82
Shashi13	47.70	47.05	47.43	51.19
Shashi4	47.70	46.84	47.69	50.93
Shashi11	47.60	46.71	47.69	50.93
Tianmen51	47.80	46.68	48.00	50.80
Tianmen35	47.62	46.90	46.67	51.84
Tianmen39	47.82	47.03	47.07	51.92
TJJ16	47.26	45.87	47.87	51.52
Q32	47.91	46.85	48.00	50.67
**Mean ±**	47.52	45.92	49.15	49.88
**STD**	0.25	0.65	0.92	0.88
**South Korean Clade**	**Overall**	**L-segment**	**M-segment**	**S-segment**
Aa08-1111	47.87	46.66	48.34	50.36
Aa09-189	47.94	46.81	48.32	50.42
LR1	47.49	45.91	48.88	49.68
76-118	47.29	45.85	48.32	49.50
JS10	47.32	45.80	48.53	49.47
76-118-POR	47.27	45.78	48.33	49.76
cl-1	47.31	45.84	48.34	49.76
Aa14-266	46.97	45.56	48.30	48.15
Aa14-272	47.08	45.74	48.51	47.85
Aa17-422	47.36	45.84	48.42	48.58
Aa05-241	47.31	45.26	49.71	50.50
Aa05-246	47.37	45.24	49.75	50.64
Aa14-172	47.32	45.21	49.80	50.85
Aa10-434	47.43	45.52	49.74	49.84
Aa10-569	47.53	45.68	49.86	49.69

Aa10-518	47.54	45.68	49.84	49.69
Aa10-521	47.53	45.64	49.86	49.69
ROKA14-11	47.61	45.91	49.92	49.46
Aa14-204	47.42	45.48	49.94	50.19
ROKA17-3	47.40	45.49	49.73	49.96
Aa17-8-R	47.36	45.42	49.68	50.30
ROKA17-8	47.52	45.60	49.97	50.18
Aa17-7	47.43	45.56	49.72	50.38
ROKA17-7	47.44	45.60	49.73	50.11
Aa16-178	47.48	45.54	49.98	49.94
Aa16-181-P	47.41	45.40	49.90	50.16
Aa14-207	47.42	45.36	49.99	50.19
Aa10-123	47.33	45.26	49.92	50.19
Aa10-265	47.40	45.34	49.86	50.47
Aa10-288	47.38	45.34	49.91	50.19
Aa10-679	47.33	45.25	49.87	50.19
Aa03-386	47.41	45.68	49.52	49.40
Aa03-161	47.45	45.68	49.53	49.67
Aa05-331	47.49	45.74	49.56	49.67
Aa13-3	47.55	45.91	49.41	49.81
Aa16-50-R	47.31	45.49	49.49	50.11
Aa14-362	47.34	45.58	49.55	48.67
Aa04-722	47.29	45.47	49.61	48.64
Aa17-66-P	47.28	45.55	49.73	48.54
Aa16-21-P	47.35	45.70	49.52	48.93
Aa16-22-P	47.22	45.42	49.51	49.00
Aa09-410	47.32	45.61	49.61	48.87
Aa09-948	47.34	45.61	49.65	48.87
Aa09-17	47.36	45.63	49.61	48.93
Aa16-19-P	47.44	45.76	49.75	49.05
Aa16-19-R	47.30	45.61	49.59	48.95
Aa14-423	47.61	45.16	50.07	50.23
Aa14-406	47.67	45.42	49.82	49.96
Aa14-404	47.69	45.45	49.82	49.96
Aa18-164	47.71	45.64	49.71	50.22
Aa18-179	47.79	45.77	49.84	49.92
Aa18-185	47.77	45.68	49.89	49.96
Aa15-58	47.58	46.14	49.41	48.94
Aa14-234	47.33	45.52	49.48	48.61
Aa14-239	47.63	46.12	49.35	48.89
Aa15-84	47.61	46.02	49.38	49.24
Aa15-69	47.62	45.63	50.13	49.18
Aa15-74	47.72	45.81	50.15	49.18
Aa15-82	47.81	45.93	50.19	49.24
ROKA16-9	47.56	46.11	49.46	49.90
Aa17-53	47.63	46.15	49.60	49.81
Aa17-52-R	47.41	46.05	48.98	49.81
Aa17-53-R	47.51	46.06	49.32	49.81
Aa17-49-R	47.60	46.11	49.55	49.81
KHF	47.30	45.56	49.45	49.93
Aa17-337	47.59	45.82	49.77	49.90
Aa17-353	47.66	45.86	49.94	49.97

**Mean ±**	47.46	45.69	49.52	49.64
**STD**	0.18	0.31	0.51	0.63
**Russian Clade**	**Overall**	**L-segment**	**M-segment**	**S-segment**
Fuyuan-Aa-26	47.34	45.22	49.31	49.38
Fuyuan-Aa-145	47.55	45.53	49.34	49.61
Galkino-AA57-2002	47.31	45.07	49.46	48.84
HTN-P88	47.23	44.82	49.93	48.33
**Mean ±**	47.36	45.16	49.51	49.04
**STD**	0.12	0.26	0.25	0.50
**Chinese Clade**	**Overall**	**L-segment**	**M-segment**	**S-segment**
84FLi	47.36	46.58	46.87	51.40
Zhongxiang5	47.86	47.65	47.52	51.10
Shashi1	47.73	47.32	47.40	50.62
HV004	48.03	47.67	47.33	52.50
Tianmen1	47.94	47.45	47.45	50.42
Tianmen15	47.91	47.67	47.57	49.70
Honghu2	47.74	47.06	47.03	50.82
Shashi13	47.70	47.05	47.43	51.19
Shashi4	47.70	46.84	47.69	50.93
Shashi11	47.60	46.71	47.69	50.93
Tianmen51	47.80	46.68	48.00	50.80
Tianmen35	47.62	46.90	46.67	51.84
Tianmen39	47.82	47.03	47.07	51.92
TJJ16	47.26	45.87	47.87	51.52
Q32	47.91	46.85	48.00	50.67
**Mean ±**	47.73	47.02	47.44	51.09
**STD**	0.20	0.47	0.38	0.66

ST5. Codon adaptation index analysis (CAI) of HTNV strains.

**All Isolates**	**CAI**											
	**L-segment**			**M-segment**			**S-segment**			**Average overall**		
	**HS**	**APO**	**NC**	**HS**	**APO**	**NC**	**HS**	**APO**	**NC**	**HS**	**APO**	**NC**
Nc167	0.785	0.569	0.528	0.805	0.591	0.557	0.820	0.627	0.580	0.803	0.596	0.555
AYW89-15	0.785	0.576	0.536	0.789	0.581	0.549	0.806	0.618	0.570	0.793	0.592	0.552
Fuyuan-Aa-26	0.775	0.560	0.518	0.795	0.582	0.544	0.815	0.621	0.583	0.795	0.588	0.548
Fuyuan-Aa-145	0.773	0.558	0.516	0.794	0.581	0.544	0.811	0.613	0.574	0.793	0.584	0.545
Galkino-AA57-2002	0.777	0.562	0.520	0.795	0.584	0.549	0.815	0.622	0.585	0.796	0.589	0.551
HTN-P88	0.776	0.562	0.521	0.797	0.583	0.547	0.814	0.621	0.582	0.796	0.589	0.550
Aa08-1111	0.781	0.568	0.524	0.777	0.566	0.539	0.804	0.614	0.576	0.787	0.583	0.546
Aa09-189	0.781	0.568	0.524	0.776	0.566	0.539	0.803	0.612	0.574	0.787	0.582	0.546
LR1	0.776	0.561	0.517	0.781	0.571	0.540	0.810	0.619	0.577	0.789	0.584	0.545
76-118	0.776	0.561	0.518	0.782	0.571	0.539	0.811	0.620	0.579	0.790	0.584	0.545
JS10	0.777	0.561	0.518	0.781	0.571	0.538	0.810	0.619	0.577	0.789	0.584	0.544
76-118-POR	0.777	0.561	0.518	0.782	0.571	0.539	0.810	0.619	0.577	0.790	0.584	0.545
cl-1	0.776	0.561	0.517	0.782	0.571	0.538	0.810	0.619	0.577	0.789	0.584	0.544
Aa14-266	0.786	0.568	0.525	0.793	0.580	0.547	0.811	0.616	0.578	0.797	0.588	0.550
Aa14-272	0.787	0.570	0.527	0.793	0.579	0.547	0.809	0.614	0.577	0.796	0.588	0.550
Aa17-422	0.785	0.568	0.524	0.789	0.580	0.548	0.791	0.604	0.564	0.788	0.584	0.545

Aa05-241	0.781	0.563	0.519	0.794	0.585	0.552	0.800	0.611	0.573	0.792	0.586	0.548
Aa05-246	0.782	0.564	0.521	0.795	0.585	0.552	0.801	0.613	0.576	0.793	0.587	0.550
Aa14-172	0.781	0.564	0.520	0.797	0.585	0.553	0.802	0.614	0.577	0.793	0.588	0.550
Aa10-434	0.788	0.572	0.528	0.799	0.587	0.556	0.803	0.613	0.573	0.797	0.591	0.552
Aa10-569	0.788	0.571	0.528	0.798	0.587	0.555	0.804	0.616	0.576	0.797	0.591	0.553
Aa10-518	0.788	0.572	0.529	0.799	0.587	0.556	0.804	0.616	0.576	0.797	0.592	0.554
Aa10-521	0.788	0.572	0.529	0.798	0.587	0.556	0.804	0.616	0.576	0.797	0.592	0.554
ROKA14-11	0.782	0.567	0.524	0.792	0.584	0.553	0.797	0.607	0.567	0.790	0.586	0.548
Aa14-204	0.785	0.569	0.526	0.793	0.585	0.552	0.800	0.610	0.568	0.793	0.588	0.549
ROKA17-3	0.783	0.568	0.525	0.798	0.588	0.556	0.797	0.607	0.566	0.793	0.588	0.549
Aa17-8-R	0.783	0.568	0.525	0.796	0.586	0.554	0.801	0.612	0.570	0.793	0.589	0.550
ROKA17-8	0.783	0.568	0.524	0.794	0.586	0.554	0.797	0.607	0.566	0.791	0.587	0.548
Aa17-7	0.783	0.568	0.524	0.798	0.588	0.555	0.798	0.609	0.567	0.793	0.588	0.549
ROKA17-7	0.783	0.568	0.524	0.797	0.587	0.555	0.797	0.609	0.568	0.792	0.588	0.549
Aa16-178	0.783	0.569	0.526	0.796	0.588	0.555	0.798	0.608	0.567	0.792	0.588	0.549
Aa16-181-P	0.783	0.569	0.526	0.795	0.586	0.553	0.801	0.611	0.570	0.793	0.589	0.550
Aa14-207	0.784	0.569	0.526	0.793	0.585	0.553	0.800	0.610	0.568	0.792	0.588	0.549
Aa10-123	0.784	0.569	0.526	0.794	0.586	0.553	0.801	0.611	0.569	0.793	0.589	0.549
Aa10-265	0.784	0.569	0.526	0.794	0.586	0.553	0.798	0.611	0.569	0.792	0.589	0.549
Aa10-288	0.784	0.569	0.526	0.795	0.586	0.553	0.800	0.610	0.568	0.793	0.588	0.549
Aa10-679	0.784	0.569	0.526	0.794	0.586	0.553	0.800	0.610	0.568	0.793	0.588	0.549
Aa03-386	0.781	0.565	0.523	0.794	0.583	0.551	0.803	0.613	0.573	0.793	0.587	0.549
Aa03-161	0.781	0.565	0.523	0.797	0.585	0.553	0.801	0.610	0.572	0.793	0.587	0.549
Aa05-331	0.781	0.565	0.523	0.797	0.585	0.553	0.801	0.610	0.572	0.793	0.587	0.549
Aa13-3	0.782	0.567	0.524	0.794	0.582	0.550	0.801	0.612	0.572	0.792	0.587	0.549
Aa16-50-R	0.782	0.566	0.524	0.796	0.585	0.553	0.802	0.611	0.573	0.793	0.587	0.550
Aa14-362	0.782	0.567	0.523	0.793	0.582	0.550	0.809	0.619	0.581	0.795	0.589	0.551
Aa04-722	0.784	0.567	0.523	0.792	0.582	0.550	0.809	0.617	0.576	0.795	0.589	0.550
Aa17-66-P	0.786	0.567	0.524	0.794	0.584	0.553	0.807	0.616	0.578	0.796	0.589	0.552
Aa16-21-P	0.785	0.567	0.523	0.792	0.583	0.552	0.809	0.618	0.580	0.795	0.589	0.552
Aa16-22-P	0.786	0.567	0.524	0.793	0.584	0.552	0.808	0.618	0.579	0.796	0.590	0.552
Aa09-410	0.784	0.567	0.523	0.792	0.582	0.550	0.810	0.619	0.581	0.795	0.589	0.551
Aa09-948	0.785	0.567	0.523	0.793	0.582	0.551	0.810	0.619	0.581	0.796	0.589	0.552
Aa09-17	0.786	0.567	0.524	0.792	0.582	0.550	0.810	0.621	0.583	0.796	0.590	0.552
Aa16-19-P	0.786	0.568	0.525	0.794	0.584	0.553	0.808	0.617	0.579	0.796	0.590	0.552
Aa16-19-R	0.786	0.567	0.524	0.793	0.583	0.552	0.810	0.619	0.582	0.796	0.590	0.553
Aa14-423	0.780	0.561	0.516	0.789	0.580	0.548	0.804	0.619	0.584	0.791	0.587	0.549
Aa14-406	0.781	0.563	0.518	0.792	0.583	0.550	0.807	0.621	0.585	0.793	0.589	0.551
Aa14-404	0.781	0.563	0.518	0.792	0.583	0.550	0.807	0.621	0.585	0.793	0.589	0.551
Aa18-164	0.782	0.564	0.520	0.795	0.586	0.554	0.805	0.617	0.580	0.794	0.589	0.551
Aa18-179	0.781	0.564	0.520	0.794	0.585	0.553	0.806	0.619	0.581	0.794	0.589	0.551
Aa18-185	0.782	0.565	0.520	0.794	0.585	0.553	0.806	0.618	0.580	0.794	0.589	0.551
Aa15-58	0.778	0.564	0.521	0.792	0.583	0.550	0.811	0.624	0.588	0.794	0.590	0.553
Aa14-234	0.784	0.569	0.526	0.790	0.580	0.547	0.797	0.607	0.566	0.790	0.585	0.546
Aa14-239	0.784	0.569	0.525	0.790	0.579	0.545	0.797	0.607	0.565	0.790	0.585	0.545
Aa15-84	0.778	0.564	0.522	0.793	0.583	0.549	0.796	0.606	0.567	0.789	0.584	0.546
Aa15-69	0.778	0.563	0.521	0.789	0.583	0.551	0.795	0.603	0.564	0.787	0.583	0.545
Aa15-74	0.778	0.564	0.521	0.789	0.583	0.551	0.795	0.603	0.564	0.787	0.583	0.545
Aa15-82	0.778	0.564	0.521	0.789	0.583	0.551	0.796	0.606	0.567	0.788	0.584	0.546
ROKA16-9	0.783	0.568	0.526	0.795	0.589	0.555	0.801	0.611	0.572	0.793	0.589	0.551
Aa17-53	0.780	0.565	0.523	0.794	0.588	0.554	0.800	0.611	0.570	0.791	0.588	0.549
Aa17-52-R	0.779	0.563	0.521	0.792	0.583	0.549	0.800	0.611	0.570	0.790	0.586	0.547
Aa17-53-R	0.779	0.564	0.521	0.792	0.584	0.550	0.800	0.611	0.570	0.790	0.586	0.547
Aa17-49-R	0.779	0.564	0.521	0.793	0.586	0.552	0.800	0.611	0.570	0.791	0.587	0.548
KHF	0.778	0.562	0.519	0.791	0.582	0.547	0.796	0.607	0.569	0.788	0.584	0.545
Aa17-337	0.779	0.564	0.522	0.789	0.585	0.549	0.795	0.609	0.570	0.788	0.586	0.547
Aa17-353	0.779	0.565	0.523	0.789	0.585	0.549	0.796	0.609	0.571	0.788	0.586	0.548
84FLi	0.779	0.566	0.523	0.782	0.566	0.532	0.810	0.612	0.568	0.790	0.581	0.541
Zhongxiang5	0.786	0.575	0.532	0.800	0.581	0.548	0.806	0.613	0.570	0.797	0.590	0.550
Shashi1	0.787	0.577	0.535	0.796	0.575	0.542	0.805	0.612	0.570	0.796	0.588	0.549
HV004	0.791	0.579	0.537	0.803	0.577	0.542	0.810	0.618	0.576	0.801	0.591	0.552
Tianmen1	0.784	0.572	0.529	0.799	0.578	0.547	0.819	0.630	0.586	0.801	0.593	0.554

Tianmen15	0.786	0.575	0.532	0.795	0.576	0.544	0.825	0.635	0.596	0.802	0.595	0.557
Honghu2	0.784	0.571	0.527	0.798	0.576	0.544	0.818	0.628	0.586	0.800	0.592	0.552
Shashi13	0.787	0.573	0.528	0.800	0.583	0.551	0.811	0.620	0.578	0.799	0.592	0.552
Shashi4	0.784	0.570	0.526	0.798	0.580	0.546	0.813	0.621	0.577	0.798	0.590	0.550
Shashi11	0.785	0.570	0.526	0.797	0.580	0.546	0.813	0.621	0.577	0.798	0.590	0.550
Tianmen51	0.788	0.572	0.528	0.798	0.576	0.545	0.819	0.635	0.591	0.802	0.594	0.555
Tianmen35	0.786	0.573	0.530	0.798	0.573	0.540	0.811	0.619	0.578	0.798	0.588	0.549
Tianmen39	0.786	0.574	0.530	0.796	0.572	0.540	0.804	0.608	0.566	0.795	0.585	0.545
TJJ16	0.784	0.570	0.527	0.798	0.578	0.548	0.793	0.602	0.563	0.792	0.583	0.546
Q32	0.783	0.568	0.527	0.795	0.575	0.545	0.795	0.603	0.563	0.791	0.582	0.545
**Mean ±**	0.782	0.567	0.524	0.793	0.582	0.549	0.805	0.615	0.574	0.793	0.588	0.549
**STD**	0.004	0.004	0.004	0.005	0.005	0.005	0.007	0.007	0.007	0.004	0.003	0.003
**South Korean Clade**	**L-segment**			**M-segment**			**S-segment**			**Average All**		
	**HS**	**APO**	**NC**	**HS**	**APO**	**NC**	**HS**	**APO**	**NC**	**HS**	**APO**	**NC**
Aa08-1111	0.781	0.568	0.524	0.777	0.566	0.539	0.804	0.614	0.576	0.787	0.583	0.546
Aa09-189	0.781	0.568	0.524	0.776	0.566	0.539	0.803	0.612	0.574	0.787	0.582	0.546
LR1	0.776	0.561	0.517	0.781	0.571	0.540	0.810	0.619	0.577	0.789	0.584	0.545
76-118	0.776	0.561	0.518	0.782	0.571	0.539	0.811	0.620	0.579	0.790	0.584	0.545
JS10	0.777	0.561	0.518	0.781	0.571	0.538	0.810	0.619	0.577	0.789	0.584	0.544
76-118-POR	0.777	0.561	0.518	0.782	0.571	0.539	0.810	0.619	0.577	0.790	0.584	0.545
cl-1	0.776	0.561	0.517	0.782	0.571	0.538	0.810	0.619	0.577	0.789	0.584	0.544
Aa14-266	0.786	0.568	0.525	0.793	0.580	0.547	0.811	0.616	0.578	0.797	0.588	0.550
Aa14-272	0.787	0.570	0.527	0.793	0.579	0.547	0.809	0.614	0.577	0.796	0.588	0.550
Aa17-422	0.785	0.568	0.524	0.789	0.580	0.548	0.791	0.604	0.564	0.788	0.584	0.545
Aa05-241	0.781	0.563	0.519	0.794	0.585	0.552	0.800	0.611	0.573	0.792	0.586	0.548
Aa05-246	0.782	0.564	0.521	0.795	0.585	0.552	0.801	0.613	0.576	0.793	0.587	0.550
Aa14-172	0.781	0.564	0.520	0.797	0.585	0.553	0.802	0.614	0.577	0.793	0.588	0.550
Aa10-434	0.788	0.572	0.528	0.799	0.587	0.556	0.803	0.613	0.573	0.797	0.591	0.552
Aa10-569	0.788	0.571	0.528	0.798	0.587	0.555	0.804	0.616	0.576	0.797	0.591	0.553
Aa10-518	0.788	0.572	0.529	0.799	0.587	0.556	0.804	0.616	0.576	0.797	0.592	0.554
Aa10-521	0.788	0.572	0.529	0.798	0.587	0.556	0.804	0.616	0.576	0.797	0.592	0.554
ROKA14-11	0.782	0.567	0.524	0.792	0.584	0.553	0.797	0.607	0.567	0.790	0.586	0.548
Aa14-204	0.785	0.569	0.526	0.793	0.585	0.552	0.800	0.610	0.568	0.793	0.588	0.549
ROKA17-3	0.783	0.568	0.525	0.798	0.588	0.556	0.797	0.607	0.566	0.793	0.588	0.549
Aa17-8-R	0.783	0.568	0.525	0.796	0.586	0.554	0.801	0.612	0.570	0.793	0.589	0.550
ROKA17-8	0.783	0.568	0.524	0.794	0.586	0.554	0.797	0.607	0.566	0.791	0.587	0.548
Aa17-7	0.783	0.568	0.524	0.798	0.588	0.555	0.798	0.609	0.567	0.793	0.588	0.549
ROKA17-7	0.783	0.568	0.524	0.797	0.587	0.555	0.797	0.609	0.568	0.792	0.588	0.549
Aa16-178	0.783	0.569	0.526	0.796	0.588	0.555	0.798	0.608	0.567	0.792	0.588	0.549
Aa16-181-P	0.783	0.569	0.526	0.795	0.586	0.553	0.801	0.611	0.570	0.793	0.589	0.550
Aa14-207	0.784	0.569	0.526	0.793	0.585	0.553	0.800	0.610	0.568	0.792	0.588	0.549
Aa10-123	0.784	0.569	0.526	0.794	0.586	0.553	0.801	0.611	0.569	0.793	0.589	0.549
Aa10-265	0.784	0.569	0.526	0.794	0.586	0.553	0.798	0.611	0.569	0.792	0.589	0.549
Aa10-288	0.784	0.569	0.526	0.795	0.586	0.553	0.800	0.610	0.568	0.793	0.588	0.549
Aa10-679	0.784	0.569	0.526	0.794	0.586	0.553	0.800	0.610	0.568	0.793	0.588	0.549
Aa03-386	0.781	0.565	0.523	0.794	0.583	0.551	0.803	0.613	0.573	0.793	0.587	0.549
Aa03-161	0.781	0.565	0.523	0.797	0.585	0.553	0.801	0.610	0.572	0.793	0.587	0.549
Aa05-331	0.781	0.565	0.523	0.797	0.585	0.553	0.801	0.610	0.572	0.793	0.587	0.549
Aa13-3	0.782	0.567	0.524	0.794	0.582	0.550	0.801	0.612	0.572	0.792	0.587	0.549
Aa16-50-R	0.782	0.566	0.524	0.796	0.585	0.553	0.802	0.611	0.573	0.793	0.587	0.550
Aa14-362	0.782	0.567	0.523	0.793	0.582	0.550	0.809	0.619	0.581	0.795	0.589	0.551
Aa04-722	0.784	0.567	0.523	0.792	0.582	0.550	0.809	0.617	0.576	0.795	0.589	0.550
Aa17-66-P	0.786	0.567	0.524	0.794	0.584	0.553	0.807	0.616	0.578	0.796	0.589	0.552
Aa16-21-P	0.785	0.567	0.523	0.792	0.583	0.552	0.809	0.618	0.580	0.795	0.589	0.552
Aa16-22-P	0.786	0.567	0.524	0.793	0.584	0.552	0.808	0.618	0.579	0.796	0.590	0.552
Aa09-410	0.784	0.567	0.523	0.792	0.582	0.550	0.810	0.619	0.581	0.795	0.589	0.551
Aa09-948	0.785	0.567	0.523	0.793	0.582	0.551	0.810	0.619	0.581	0.796	0.589	0.552
Aa09-17	0.786	0.567	0.524	0.792	0.582	0.550	0.810	0.621	0.583	0.796	0.590	0.552
Aa16-19-P	0.786	0.568	0.525	0.794	0.584	0.553	0.808	0.617	0.579	0.796	0.590	0.552
Aa16-19-R	0.786	0.567	0.524	0.793	0.583	0.552	0.810	0.619	0.582	0.796	0.590	0.553

Aa14-423	0.780	0.561	0.516	0.789	0.580	0.548	0.804	0.619	0.584	0.791	0.587	0.549
Aa14-406	0.781	0.563	0.518	0.792	0.583	0.550	0.807	0.621	0.585	0.793	0.589	0.551
Aa14-404	0.781	0.563	0.518	0.792	0.583	0.550	0.807	0.621	0.585	0.793	0.589	0.551
Aa18-164	0.782	0.564	0.520	0.795	0.586	0.554	0.805	0.617	0.580	0.794	0.589	0.551
Aa18-179	0.781	0.564	0.520	0.794	0.585	0.553	0.806	0.619	0.581	0.794	0.589	0.551
Aa18-185	0.782	0.565	0.520	0.794	0.585	0.553	0.806	0.618	0.580	0.794	0.589	0.551
Aa15-58	0.778	0.564	0.521	0.792	0.583	0.550	0.811	0.624	0.588	0.794	0.590	0.553
Aa14-234	0.784	0.569	0.526	0.790	0.580	0.547	0.797	0.607	0.566	0.790	0.585	0.546
Aa14-239	0.784	0.569	0.525	0.790	0.579	0.545	0.797	0.607	0.565	0.790	0.585	0.545
Aa15-84	0.778	0.564	0.522	0.793	0.583	0.549	0.796	0.606	0.567	0.789	0.584	0.546
Aa15-69	0.778	0.563	0.521	0.789	0.583	0.551	0.795	0.603	0.564	0.787	0.583	0.545
Aa15-74	0.778	0.564	0.521	0.789	0.583	0.551	0.795	0.603	0.564	0.787	0.583	0.545
Aa15-82	0.778	0.564	0.521	0.789	0.583	0.551	0.796	0.606	0.567	0.788	0.584	0.546
ROKA16-9	0.783	0.568	0.526	0.795	0.589	0.555	0.801	0.611	0.572	0.793	0.589	0.551
Aa17-53	0.780	0.565	0.523	0.794	0.588	0.554	0.800	0.611	0.570	0.791	0.588	0.549
Aa17-52-R	0.779	0.563	0.521	0.792	0.583	0.549	0.800	0.611	0.570	0.790	0.586	0.547
Aa17-53-R	0.779	0.564	0.521	0.792	0.584	0.550	0.800	0.611	0.570	0.790	0.586	0.547
Aa17-49-R	0.779	0.564	0.521	0.793	0.586	0.552	0.800	0.611	0.570	0.791	0.587	0.548
KHF	0.778	0.562	0.519	0.791	0.582	0.547	0.796	0.607	0.569	0.788	0.584	0.545
Aa17-337	0.779	0.564	0.522	0.789	0.585	0.549	0.795	0.609	0.570	0.788	0.586	0.547
Aa17-353	0.779	0.565	0.523	0.789	0.585	0.549	0.796	0.609	0.571	0.788	0.586	0.548
**Mean ±**	0.782	0.566	0.523	0.792	0.583	0.550	0.803	0.613	0.574	0.792	0.587	0.549
**STD**	0.003	0.003	0.003	0.005	0.005	0.005	0.005	0.005	0.006	0.003	0.002	0.003
**Russian Clade**	**L-segment**			**M-segment**			**S-segment**			**Average All**		
	**HS**	**APO**	**NC**	**HS**	**APO**	**NC**	**HS**	**APO**	**NC**	**HS**	**APO**	**NC**
Fuyuan-Aa-26	0.775	0.560	0.518	0.795	0.582	0.544	0.815	0.621	0.583	0.795	0.588	0.548
Fuyuan-Aa-145	0.773	0.558	0.516	0.794	0.581	0.544	0.811	0.613	0.574	0.793	0.584	0.545
Galkino-AA57-2002	0.777	0.562	0.520	0.795	0.584	0.549	0.815	0.622	0.585	0.796	0.589	0.551
HTN-P88	0.776	0.562	0.521	0.797	0.583	0.547	0.814	0.621	0.582	0.796	0.589	0.550
**Mean ±**	0.775	0.561	0.519	0.795	0.583	0.546	0.814	0.619	0.581	0.795	0.588	0.549
**STD**	0.001	0.002	0.002	0.001	0.001	0.002	0.002	0.004	0.004	0.001	0.002	0.002
**Chinese Clade**	**L-segment**			**M-segment**			**S-segment**			**Average All**		
	**HS**	**APO**	**NC**	**HS**	**APO**	**NC**	**HS**	**APO**	**NC**	**HS**	**APO**	**NC**
84FLi	0.779	0.566	0.523	0.782	0.566	0.532	0.810	0.612	0.568	0.790	0.581	0.541
Zhongxiang5	0.786	0.575	0.532	0.800	0.581	0.548	0.806	0.613	0.570	0.797	0.590	0.550
Shashi1	0.787	0.577	0.535	0.796	0.575	0.542	0.805	0.612	0.570	0.796	0.588	0.549
HV004	0.791	0.579	0.537	0.803	0.577	0.542	0.810	0.618	0.576	0.801	0.591	0.552
Tianmen1	0.784	0.572	0.529	0.799	0.578	0.547	0.819	0.630	0.586	0.801	0.593	0.554
Tianmen15	0.786	0.575	0.532	0.795	0.576	0.544	0.825	0.635	0.596	0.802	0.595	0.557
Honghu2	0.784	0.571	0.527	0.798	0.576	0.544	0.818	0.628	0.586	0.800	0.592	0.552
Shashi13	0.787	0.573	0.528	0.800	0.583	0.551	0.811	0.620	0.578	0.799	0.592	0.552
Shashi4	0.784	0.570	0.526	0.798	0.580	0.546	0.813	0.621	0.577	0.798	0.590	0.550
Shashi11	0.785	0.570	0.526	0.797	0.580	0.546	0.813	0.621	0.577	0.798	0.590	0.550
Tianmen51	0.788	0.572	0.528	0.798	0.576	0.545	0.819	0.635	0.591	0.802	0.594	0.555
Tianmen35	0.786	0.573	0.530	0.798	0.573	0.540	0.811	0.619	0.578	0.798	0.588	0.549
Tianmen39	0.786	0.574	0.530	0.796	0.572	0.540	0.804	0.608	0.566	0.795	0.585	0.545
TJJ16	0.784	0.570	0.527	0.798	0.578	0.548	0.793	0.602	0.563	0.792	0.583	0.546
Q32	0.783	0.568	0.527	0.795	0.575	0.545	0.795	0.603	0.563	0.791	0.582	0.545
**Mean ±**	0.785	0.572	0.529	0.797	0.576	0.544	0.810	0.618	0.576	0.797	0.589	0.550
**STD**	0.003	0.003	0.004	0.004	0.004	0.004	0.008	0.010	0.010	0.004	0.004	0.004

ST6. Relative codon deoptimization index analysis (RCDI) of HTNV strains.

All Isolates	RCDI											
	L-segment			M-segment			S-segment			Average All		
	HS	AP0	NC	HS	AP0	NC	HS	AP0	NC	HS	AP0	NC
Nc167	1.364	1.830	2.148	1.229	1.532	1.681	1.208	1.447	1.599	1.267	1.603	1.809
AYW89-15	1.359	1.759	2.038	1.285	1.573	1.711	1.317	1.564	1.728	1.320	1.632	1.826
Fuyuan-Aa-26	1.426	1.873	2.166	1.285	1.600	1.781	1.263	1.521	1.671	1.325	1.665	1.873
Fuyuan-Aa-145	1.427	1.885	2.197	1.286	1.602	1.775	1.252	1.520	1.675	1.322	1.669	1.882
Galkino/AA57/2002	1.420	1.857	2.129	1.274	1.582	1.743	1.273	1.524	1.652	1.322	1.654	1.841
HTN-P88	1.422	1.857	2.123	1.271	1.595	1.776	1.289	1.549	1.690	1.327	1.667	1.863
Aa08-1111	1.370	1.797	2.093	1.353	1.665	1.786	1.306	1.572	1.711	1.343	1.678	1.863
Aa09-189	1.365	1.789	2.081	1.354	1.666	1.787	1.306	1.574	1.715	1.342	1.676	1.861
LR1	1.404	1.865	2.195	1.335	1.649	1.802	1.307	1.578	1.762	1.349	1.697	1.920
76-118	1.407	1.865	2.189	1.336	1.653	1.805	1.302	1.573	1.753	1.348	1.697	1.916
JS10	1.406	1.865	2.189	1.334	1.649	1.804	1.306	1.579	1.759	1.349	1.698	1.917
76-118/POR	1.407	1.866	2.190	1.337	1.653	1.806	1.304	1.576	1.757	1.349	1.698	1.918
cl-1	1.407	1.865	2.192	1.337	1.653	1.806	1.304	1.574	1.756	1.349	1.697	1.918
Aa14-266	1.396	1.841	2.139	1.313	1.629	1.788	1.328	1.610	1.755	1.346	1.693	1.894
Aa14-272	1.389	1.831	2.129	1.316	1.636	1.794	1.333	1.615	1.758	1.346	1.694	1.894
Aa17-422	1.403	1.866	2.203	1.312	1.606	1.743	1.336	1.609	1.773	1.350	1.694	1.906
Aa05-241	1.426	1.892	2.224	1.287	1.594	1.763	1.298	1.551	1.685	1.337	1.679	1.891
Aa05-246	1.421	1.880	2.203	1.284	1.590	1.758	1.293	1.544	1.674	1.333	1.671	1.878
Aa14-172	1.427	1.891	2.221	1.277	1.592	1.757	1.292	1.537	1.656	1.332	1.673	1.878
Aa10-434	1.390	1.842	2.167	1.270	1.574	1.727	1.311	1.577	1.736	1.324	1.664	1.877
Aa10-569	1.386	1.834	2.154	1.274	1.586	1.754	1.311	1.570	1.722	1.324	1.663	1.877
Aa10-518	1.385	1.831	2.149	1.271	1.576	1.728	1.311	1.570	1.722	1.322	1.659	1.866
Aa10-521	1.385	1.831	2.150	1.273	1.574	1.726	1.311	1.570	1.722	1.323	1.658	1.866
ROKA14-11	1.398	1.857	2.196	1.282	1.567	1.698	1.333	1.613	1.785	1.338	1.679	1.893
Aa14-204	1.402	1.857	2.192	1.285	1.583	1.744	1.303	1.579	1.760	1.330	1.673	1.899
ROKA17-3	1.399	1.847	2.171	1.280	1.578	1.736	1.316	1.593	1.772	1.332	1.673	1.893
Aa17-8-R	1.404	1.856	2.185	1.287	1.591	1.750	1.300	1.571	1.753	1.330	1.673	1.896
ROKA17-8	1.399	1.852	2.180	1.280	1.569	1.719	1.317	1.595	1.776	1.332	1.672	1.892
Aa17-7	1.399	1.851	2.179	1.282	1.581	1.737	1.303	1.575	1.759	1.328	1.669	1.892
ROKA17-7	1.399	1.852	2.180	1.284	1.583	1.740	1.312	1.583	1.761	1.332	1.673	1.894
Aa16-178	1.402	1.849	2.174	1.275	1.562	1.705	1.315	1.597	1.778	1.331	1.669	1.886
Aa16-181-P	1.406	1.853	2.178	1.283	1.579	1.734	1.308	1.582	1.764	1.332	1.671	1.892
Aa14-207	1.406	1.858	2.191	1.282	1.575	1.728	1.303	1.579	1.760	1.330	1.671	1.893
Aa10-123	1.410	1.869	2.208	1.283	1.578	1.730	1.301	1.573	1.754	1.331	1.673	1.897
Aa10-265	1.403	1.847	2.167	1.284	1.579	1.731	1.295	1.552	1.716	1.327	1.659	1.871
Aa10-288	1.404	1.854	2.178	1.281	1.576	1.730	1.303	1.579	1.760	1.329	1.670	1.889
Aa10-679	1.408	1.857	2.183	1.284	1.577	1.730	1.303	1.579	1.760	1.332	1.671	1.891
Aa03-386	1.406	1.861	2.182	1.298	1.593	1.735	1.325	1.596	1.756	1.343	1.683	1.891
Aa03-161	1.404	1.854	2.165	1.290	1.601	1.758	1.325	1.599	1.754	1.340	1.685	1.892
Aa05-331	1.402	1.850	2.161	1.289	1.599	1.756	1.325	1.599	1.754	1.339	1.683	1.890
Aa13-3	1.398	1.846	2.157	1.298	1.606	1.756	1.314	1.578	1.739	1.337	1.677	1.884
Aa16-50-R	1.410	1.867	2.183	1.293	1.596	1.746	1.313	1.585	1.737	1.339	1.683	1.889
Aa14-362	1.406	1.858	2.188	1.299	1.602	1.754	1.318	1.584	1.732	1.341	1.681	1.891
Aa04-722	1.405	1.862	2.193	1.299	1.598	1.741	1.332	1.623	1.815	1.345	1.694	1.916
Aa17-66-P	1.402	1.864	2.197	1.290	1.582	1.713	1.323	1.601	1.761	1.338	1.682	1.890
Aa16-21-P	1.404	1.867	2.202	1.298	1.592	1.730	1.319	1.603	1.769	1.340	1.687	1.900
Aa16-22-P	1.411	1.888	2.235	1.298	1.591	1.731	1.313	1.588	1.738	1.341	1.689	1.901
Aa09-410	1.406	1.869	2.204	1.299	1.602	1.749	1.314	1.586	1.748	1.340	1.686	1.900
Aa09-948	1.406	1.875	2.217	1.297	1.600	1.747	1.314	1.586	1.748	1.339	1.687	1.904
Aa09-17	1.405	1.873	2.215	1.299	1.602	1.749	1.311	1.579	1.737	1.338	1.685	1.900
Aa16-19-P	1.397	1.858	2.191	1.290	1.581	1.709	1.311	1.588	1.737	1.333	1.676	1.879
Aa16-19-R	1.405	1.874	2.215	1.295	1.591	1.727	1.310	1.585	1.733	1.337	1.683	1.892
Aa14-423	1.427	1.912	2.267	1.291	1.586	1.733	1.304	1.554	1.689	1.341	1.684	1.896
Aa14-406	1.410	1.881	2.219	1.285	1.579	1.723	1.304	1.558	1.696	1.333	1.673	1.879
Aa14-404	1.410	1.883	2.221	1.285	1.579	1.723	1.304	1.558	1.696	1.333	1.673	1.880
Aa18-164	1.404	1.870	2.208	1.279	1.569	1.709	1.303	1.570	1.714	1.329	1.670	1.877
Aa18-179	1.401	1.867	2.203	1.277	1.568	1.708	1.304	1.560	1.704	1.327	1.665	1.872

Aa18-185	1.401	1.867	2.203	1.275	1.564	1.705	1.305	1.563	1.709	1.327	1.665	1.872
Aa15-58	1.399	1.846	2.158	1.305	1.608	1.771	1.316	1.566	1.704	1.340	1.673	1.878
Aa14-234	1.401	1.857	2.184	1.309	1.626	1.807	1.340	1.624	1.786	1.350	1.702	1.926
Aa14-239	1.385	1.833	2.149	1.311	1.637	1.820	1.328	1.608	1.772	1.341	1.693	1.914
Aa15-84	1.397	1.839	2.143	1.302	1.616	1.799	1.332	1.622	1.792	1.344	1.692	1.911
Aa15-69	1.405	1.854	2.159	1.294	1.592	1.761	1.339	1.644	1.817	1.346	1.697	1.912
Aa15-74	1.400	1.849	2.154	1.290	1.586	1.754	1.339	1.644	1.817	1.343	1.693	1.908
Aa15-82	1.399	1.843	2.147	1.292	1.588	1.757	1.332	1.622	1.792	1.341	1.684	1.899
ROKA16-9	1.387	1.821	2.112	1.300	1.581	1.740	1.311	1.568	1.711	1.333	1.657	1.854
Aa17-53	1.395	1.844	2.149	1.295	1.572	1.725	1.311	1.575	1.735	1.334	1.664	1.870
Aa17-52-R	1.401	1.850	2.154	1.319	1.618	1.783	1.311	1.575	1.735	1.344	1.681	1.891
Aa17-53-R	1.400	1.850	2.153	1.311	1.610	1.784	1.311	1.575	1.735	1.341	1.678	1.891
Aa17-49-R	1.398	1.843	2.140	1.301	1.589	1.753	1.311	1.575	1.735	1.337	1.669	1.876
KHF	1.419	1.880	2.206	1.315	1.618	1.788	1.317	1.594	1.738	1.350	1.697	1.911
Aa17-337	1.405	1.844	2.136	1.307	1.589	1.753	1.314	1.579	1.721	1.342	1.671	1.870
Aa17-353	1.402	1.833	2.115	1.302	1.583	1.750	1.313	1.579	1.719	1.339	1.665	1.861
84FLi	1.377	1.808	2.082	1.346	1.706	1.875	1.267	1.558	1.725	1.330	1.691	1.894
Zhongxiang5	1.338	1.741	2.011	1.300	1.666	1.845	1.230	1.482	1.611	1.289	1.630	1.822
Shashi1	1.333	1.722	1.977	1.302	1.658	1.816	1.263	1.537	1.721	1.299	1.639	1.838
HV004	1.330	1.736	2.008	1.302	1.700	1.911	1.234	1.473	1.616	1.289	1.636	1.845
Tianmen1	1.356	1.771	2.055	1.302	1.659	1.816	1.224	1.451	1.587	1.294	1.627	1.819
Tianmen15	1.353	1.769	2.072	1.297	1.657	1.827	1.244	1.478	1.604	1.298	1.635	1.834
Honghu2	1.367	1.800	2.115	1.304	1.666	1.823	1.234	1.478	1.612	1.302	1.648	1.850
Shashi13	1.371	1.819	2.156	1.299	1.632	1.782	1.252	1.521	1.696	1.307	1.657	1.878
Shashi4	1.376	1.821	2.146	1.290	1.623	1.778	1.238	1.503	1.693	1.301	1.649	1.872
Shashi11	1.382	1.833	2.169	1.291	1.625	1.783	1.238	1.503	1.693	1.304	1.654	1.882
Tianmen51	1.375	1.834	2.175	1.281	1.651	1.819	1.216	1.423	1.561	1.291	1.636	1.852
Tianmen35	1.363	1.792	2.085	1.321	1.715	1.896	1.233	1.478	1.629	1.306	1.662	1.870
Tianmen39	1.364	1.795	2.101	1.312	1.699	1.870	1.242	1.509	1.668	1.306	1.668	1.880
TJJ16	1.390	1.828	2.124	1.309	1.688	1.856	1.238	1.472	1.583	1.312	1.663	1.854
Q32	1.369	1.810	2.103	1.317	1.702	1.880	1.236	1.486	1.604	1.307	1.666	1.862
**Mean ±**	1.395	1.844	2.160	1.297	1.609	1.765	1.297	1.564	1.721	1.330	1.672	1.882
**STD**	0.020	0.035	0.052	0.020	0.038	0.046	0.032	0.044	0.055	0.017	0.019	0.024

**South Korean Clade**	**L-segment**			**M-segment**			**S-segment**			**Average All**		
	**HS**	**AP**	**NC**	**HS**	**AP**	**NC**	**HS**	**AP**	**NC**	**HS**	**AP**	**NC**
Aa08-1111	1.370	1.797	2.093	1.353	1.665	1.786	1.306	1.572	1.711	1.343	1.678	1.863
Aa09-189	1.365	1.789	2.081	1.354	1.666	1.787	1.306	1.574	1.715	1.342	1.676	1.861
LR1	1.404	1.865	2.195	1.335	1.649	1.802	1.307	1.578	1.762	1.349	1.697	1.920
76-118	1.407	1.865	2.189	1.336	1.653	1.805	1.302	1.573	1.753	1.348	1.697	1.916
JS10	1.406	1.865	2.189	1.334	1.649	1.804	1.306	1.579	1.759	1.349	1.698	1.917
76-118-POR	1.407	1.866	2.190	1.337	1.653	1.806	1.304	1.576	1.757	1.349	1.698	1.918
cl-1	1.407	1.865	2.192	1.337	1.653	1.806	1.304	1.574	1.756	1.349	1.697	1.918
Aa14-266	1.396	1.841	2.139	1.313	1.629	1.788	1.328	1.610	1.755	1.346	1.693	1.894
Aa14-272	1.389	1.831	2.129	1.316	1.636	1.794	1.333	1.615	1.758	1.346	1.694	1.894
Aa17-422	1.403	1.866	2.203	1.312	1.606	1.743	1.336	1.609	1.773	1.350	1.694	1.906
Aa05-241	1.426	1.892	2.224	1.287	1.594	1.763	1.298	1.551	1.685	1.337	1.679	1.891
Aa05-246	1.421	1.880	2.203	1.284	1.590	1.758	1.293	1.544	1.674	1.333	1.671	1.878
Aa14-172	1.427	1.891	2.221	1.277	1.592	1.757	1.292	1.537	1.656	1.332	1.673	1.878
Aa10-434	1.390	1.842	2.167	1.270	1.574	1.727	1.311	1.577	1.736	1.324	1.664	1.877
Aa10-569	1.386	1.834	2.154	1.274	1.586	1.754	1.311	1.570	1.722	1.324	1.663	1.877
Aa10-518	1.385	1.831	2.149	1.271	1.576	1.728	1.311	1.570	1.722	1.322	1.659	1.866
Aa10-521	1.385	1.831	2.150	1.273	1.574	1.726	1.311	1.570	1.722	1.323	1.658	1.866
ROKA14-11	1.398	1.857	2.196	1.282	1.567	1.698	1.333	1.613	1.785	1.338	1.679	1.893
Aa14-204	1.402	1.857	2.192	1.285	1.583	1.744	1.303	1.579	1.760	1.330	1.673	1.899
ROKA17-3	1.399	1.847	2.171	1.280	1.578	1.736	1.316	1.593	1.772	1.332	1.673	1.893
Aa17-8-R	1.404	1.856	2.185	1.287	1.591	1.750	1.300	1.571	1.753	1.330	1.673	1.896
ROKA17-8	1.399	1.852	2.180	1.280	1.569	1.719	1.317	1.595	1.776	1.332	1.672	1.892
Aa17-7	1.399	1.851	2.179	1.282	1.581	1.737	1.303	1.575	1.759	1.328	1.669	1.892
ROKA17-7	1.399	1.852	2.180	1.284	1.583	1.740	1.312	1.583	1.761	1.332	1.673	1.894
Aa16-178	1.402	1.849	2.174	1.275	1.562	1.705	1.315	1.597	1.778	1.331	1.669	1.886

Aa16-181-P	1.406	1.853	2.178	1.283	1.579	1.734	1.308	1.582	1.764	1.332	1.671	1.892
Aa14-207	1.406	1.858	2.191	1.282	1.575	1.728	1.303	1.579	1.760	1.330	1.671	1.893
Aa10-123	1.410	1.869	2.208	1.283	1.578	1.730	1.301	1.573	1.754	1.331	1.673	1.897
Aa10-265	1.403	1.847	2.167	1.284	1.579	1.731	1.295	1.552	1.716	1.327	1.659	1.871
Aa10-288	1.404	1.854	2.178	1.281	1.576	1.730	1.303	1.579	1.760	1.329	1.670	1.889
Aa10-679	1.408	1.857	2.183	1.284	1.577	1.730	1.303	1.579	1.760	1.332	1.671	1.891
Aa03-386	1.406	1.861	2.182	1.298	1.593	1.735	1.325	1.596	1.756	1.343	1.683	1.891
Aa03-161	1.404	1.854	2.165	1.290	1.601	1.758	1.325	1.599	1.754	1.340	1.685	1.892
Aa05-331	1.402	1.850	2.161	1.289	1.599	1.756	1.325	1.599	1.754	1.339	1.683	1.890
Aa13-3	1.398	1.846	2.157	1.298	1.606	1.756	1.314	1.578	1.739	1.337	1.677	1.884
Aa16-50-R	1.410	1.867	2.183	1.293	1.596	1.746	1.313	1.585	1.737	1.339	1.683	1.889
Aa14-362	1.406	1.858	2.188	1.299	1.602	1.754	1.318	1.584	1.732	1.341	1.681	1.891
Aa04-722	1.405	1.862	2.193	1.299	1.598	1.741	1.332	1.623	1.815	1.345	1.694	1.916
Aa17-66-P	1.402	1.864	2.197	1.290	1.582	1.713	1.323	1.601	1.761	1.338	1.682	1.890
Aa16-21-P	1.404	1.867	2.202	1.298	1.592	1.730	1.319	1.603	1.769	1.340	1.687	1.900
Aa16-22-P	1.411	1.888	2.235	1.298	1.591	1.731	1.313	1.588	1.738	1.341	1.689	1.901
Aa09-410	1.406	1.869	2.204	1.299	1.602	1.749	1.314	1.586	1.748	1.340	1.686	1.900
Aa09-948	1.406	1.875	2.217	1.297	1.600	1.747	1.314	1.586	1.748	1.339	1.687	1.904
Aa09-17	1.405	1.873	2.215	1.299	1.602	1.749	1.311	1.579	1.737	1.338	1.685	1.900
Aa16-19-P	1.397	1.858	2.191	1.290	1.581	1.709	1.311	1.588	1.737	1.333	1.676	1.879
Aa16-19-R	1.405	1.874	2.215	1.295	1.591	1.727	1.310	1.585	1.733	1.337	1.683	1.892
Aa14-423	1.427	1.912	2.267	1.291	1.586	1.733	1.304	1.554	1.689	1.341	1.684	1.896
Aa14-406	1.410	1.881	2.219	1.285	1.579	1.723	1.304	1.558	1.696	1.333	1.673	1.879
Aa14-404	1.410	1.883	2.221	1.285	1.579	1.723	1.304	1.558	1.696	1.333	1.673	1.880
Aa18-164	1.404	1.870	2.208	1.279	1.569	1.709	1.303	1.570	1.714	1.329	1.670	1.877
Aa18-179	1.401	1.867	2.203	1.277	1.568	1.708	1.304	1.560	1.704	1.327	1.665	1.872
Aa18-185	1.401	1.867	2.203	1.275	1.564	1.705	1.305	1.563	1.709	1.327	1.665	1.872
Aa15-58	1.399	1.846	2.158	1.305	1.608	1.771	1.316	1.566	1.704	1.340	1.673	1.878
Aa14-234	1.401	1.857	2.184	1.309	1.626	1.807	1.340	1.624	1.786	1.350	1.702	1.926
Aa14-239	1.385	1.833	2.149	1.311	1.637	1.820	1.328	1.608	1.772	1.341	1.693	1.914
Aa15-84	1.397	1.839	2.143	1.302	1.616	1.799	1.332	1.622	1.792	1.344	1.692	1.911
Aa15-69	1.405	1.854	2.159	1.294	1.592	1.761	1.339	1.644	1.817	1.346	1.697	1.912
Aa15-74	1.400	1.849	2.154	1.290	1.586	1.754	1.339	1.644	1.817	1.343	1.693	1.908
Aa15-82	1.399	1.843	2.147	1.292	1.588	1.757	1.332	1.622	1.792	1.341	1.684	1.899
ROKA16-9	1.387	1.821	2.112	1.300	1.581	1.740	1.311	1.568	1.711	1.333	1.657	1.854
Aa17-53	1.395	1.844	2.149	1.295	1.572	1.725	1.311	1.575	1.735	1.334	1.664	1.870
Aa17-52-R	1.401	1.850	2.154	1.319	1.618	1.783	1.311	1.575	1.735	1.344	1.681	1.891
Aa17-53-R	1.400	1.850	2.153	1.311	1.610	1.784	1.311	1.575	1.735	1.341	1.678	1.891
Aa17-49-R	1.398	1.843	2.140	1.301	1.589	1.753	1.311	1.575	1.735	1.337	1.669	1.876
KHF	1.419	1.880	2.206	1.315	1.618	1.788	1.317	1.594	1.738	1.350	1.697	1.911
Aa17-337	1.405	1.844	2.136	1.307	1.589	1.753	1.314	1.579	1.721	1.342	1.671	1.870
Aa17-353	1.402	1.833	2.115	1.302	1.583	1.750	1.313	1.579	1.719	1.339	1.665	1.861
**Mean ±**	1.402	1.856	2.178	1.297	1.597	1.751	1.313	1.584	1.744	1.337	1.679	1.891
**STD**	0.011	0.020	0.033	0.019	0.026	0.030	0.011	0.021	0.032	0.007	0.012	0.016
**Russian Clade**	**L-segment**			**M-segment**			**S-segment**			**Average All**		
	**HS**	**AP**	**NC**	**HS**	**AP**	**NC**	**HS**	**AP**	**NC**	**HS**	**AP**	**NC**
Fuyuan-Aa-26	1.426	1.873	2.166	1.285	1.600	1.781	1.263	1.521	1.671	1.325	1.665	1.873
Fuyuan-Aa-145	1.427	1.885	2.197	1.286	1.602	1.775	1.252	1.520	1.675	1.322	1.669	1.882
Galkino-AA57-2002	1.420	1.857	2.129	1.274	1.582	1.743	1.273	1.524	1.652	1.322	1.654	1.841
HTN-P88	1.422	1.857	2.123	1.271	1.595	1.776	1.289	1.549	1.690	1.327	1.667	1.863
**Mean ±**	1.424	1.868	2.154	1.279	1.595	1.769	1.269	1.529	1.672	1.324	1.664	1.865
**STD**	0.003	0.012	0.030	0.007	0.008	0.015	0.014	0.012	0.014	0.002	0.006	0.015
**Chinese Clade**	**L-segment**			**M-segment**			**S-segment**			**Average All**		
	**HS**	**AP**	**NC**	**HS**	**AP**	**NC**	**HS**	**AP**	**NC**	**HS**	**AP**	**NC**
84FLi	1.377	1.808	2.082	1.346	1.706	1.875	1.267	1.558	1.725	1.330	1.691	1.894
Zhongxiang5	1.338	1.741	2.011	1.300	1.666	1.845	1.230	1.482	1.611	1.289	1.630	1.822
Shashi1	1.333	1.722	1.977	1.302	1.658	1.816	1.263	1.537	1.721	1.299	1.639	1.838
HV004	1.330	1.736	2.008	1.302	1.700	1.911	1.234	1.473	1.616	1.289	1.636	1.845

Tianmen1	1.356	1.771	2.055	1.302	1.659	1.816	1.224	1.451	1.587	1.294	1.627	1.819
Tianmen15	1.353	1.769	2.072	1.297	1.657	1.827	1.244	1.478	1.604	1.298	1.635	1.834
Honghu2	1.367	1.800	2.115	1.304	1.666	1.823	1.234	1.478	1.612	1.302	1.648	1.850
Shashi13	1.371	1.819	2.156	1.299	1.632	1.782	1.252	1.521	1.696	1.307	1.657	1.878
Shashi4	1.376	1.821	2.146	1.290	1.623	1.778	1.238	1.503	1.693	1.301	1.649	1.872
Shashi11	1.382	1.833	2.169	1.291	1.625	1.783	1.238	1.503	1.693	1.304	1.654	1.882
Tianmen51	1.375	1.834	2.175	1.281	1.651	1.819	1.216	1.423	1.561	1.291	1.636	1.852
Tianmen35	1.363	1.792	2.085	1.321	1.715	1.896	1.233	1.478	1.629	1.306	1.662	1.870
Tianmen39	1.364	1.795	2.101	1.312	1.699	1.870	1.242	1.509	1.668	1.306	1.668	1.880
TJJ16	1.390	1.828	2.124	1.309	1.688	1.856	1.238	1.472	1.583	1.312	1.663	1.854
Q32	1.369	1.810	2.103	1.317	1.702	1.880	1.236	1.486	1.604	1.307	1.666	1.862
**Mean ±**	1.363	1.792	2.092	1.305	1.670	1.838	1.239	1.490	1.640	1.302	1.651	1.857
**STD**	0.017	0.035	0.058	0.015	0.029	0.041	0.013	0.032	0.052	0.010	0.017	0.022
